# Compensatory Internalization of Pma1 in V-ATPase Mutants in *Saccharomyces cerevisiae* Requires Calcium- and Glucose-Sensitive Phosphatases

**DOI:** 10.1534/genetics.117.300594

**Published:** 2017-12-18

**Authors:** Swetha Devi Velivela, Patricia M. Kane

**Affiliations:** Department of Biochemistry and Molecular Biology, State University of New York Upstate Medical University, Syracuse, New York 13210

**Keywords:** acidification, arrestin, calcineurin, endocytosis, phosphatase, proton pump, synthetic lethality, ubiquitination, vacuole

## Abstract

Loss of V-ATPase activity in organelles, whether through V-ATPase inhibition or V-ATPase (*vma*) mutations, triggers a compensatory downregulation of the essential plasma membrane proton pump Pma1 in *Saccharomyces cerevisiae*. We have previously determined that the α-arrestin Rim8 and ubiquitin ligase Rsp5 are essential for Pma1 ubiquination and endocytosis in response to loss of V-ATPase activity. Here, we show that Pma1 endocytosis in V-ATPase mutants does not require Rim101 pathway components upstream and downstream of Rim8, indicating that Rim8 is acting independently in Pma1 internalization. We find that two phosphatases, the calcium-responsive phosphatase calcineurin and the glucose-sensitive phosphatase Glc7 (PP1), and one of the Glc7 regulatory subunits Reg1, exhibit negative synthetic genetic interactions with *vma* mutants, and demonstrate that both phosphatases are essential for ubiquitination and endocytic downregulation of Pma1 in these mutants. Although both acute and chronic loss of V-ATPase activity trigger the internalization of ∼50% of surface Pma1, a comparable reduction in Pma1 expression in a *pma1-007* mutant neither compensates for loss of V-ATPase activity nor stops further Pma1 endocytosis. The results indicate that the cell surface level of Pma1 is not directly sensed and that internalized Pma1 may play a role in compensating for loss of V-ATPase-dependent acidification. Taken together, these results provide new insights into cross talk between two major proton pumps central to cellular pH control.

## Abstract (precis)

Loss of V-ATPase activity in organelles triggers compensatory endocytic downregulation of the plasma membrane proton pump Pma1. Here, Velivela and Kane...

ALL eukaryotic cells adapt to different environmental and internal stresses to survive. Cells have different signaling mechanisms to elicit specific responses to diverse stresses they encounter. One such mechanism is continuous remodeling of the plasma membrane (PM) in response to different stimuli. Yeast cells remove or downregulate several amino acid, sugar, and metal transporters from the PM through ubiquitin-mediated endocytosis in response to the availability of the specific nutrients in the external media (Lin *et al.* 2008; Nikko *et al.* 2008; Nikko and Pelham 2009; Paiva *et al.* 2009; Hatakeyama *et al.* 2010; O’Donnell *et al.* 2010; MacGurn *et al.* 2011; Becuwe and Léon 2014; Llopis-Torregrosa *et al.* 2016). Upon receiving specific internalization stimuli, these PM proteins are ubiquitinated. This step is accomplished by an E3 ubiquitin ligase, Rsp5 (homolog of the mammalian Nedd4 family ubiquitin ligases) with the help of the PY motif-containing α-arrestin family adapter proteins (Belgareh-Touzé *et al.* 2008; Lin *et al.* 2008; Shiga *et al.* 2014). The PM proteins modified by ubiquitination are internalized and ultimately delivered to the vacuolar lumen for degradation. Several of the yeast α-arrestins have been shown to act as cytosolic sensors of nutrient levels. These α-arrestins tend to be highly phosphorylated, with altered phosphorylation in response to the presence of the relevant nutrient stimulating their recruitment to the PM, interaction with the corresponding transporter, and binding to Rsp5.

We previously described a potential pH homeostatic mechanism involving the ubiquitin-mediated endocytic downregulation of the PM H^+^-ATPase (Pma1) in response to the inhibition or deletion of organellar vacuolar-H^+^-ATPases (V-ATPases) (Martinez-Munoz and Kane 2008; Tarsio *et al.* 2011; Smardon and Kane 2014). V-ATPases are multi-subunit proton pumps, which acidify the lumens of the acidic organelles such as vacuoles/lysosomes, Golgi, and endosomes in all eukaryotic cells. Pma1 is a single-subunit transmembrane proton pump, which pumps protons to the cell exterior and acts as the primary determinant of cytosolic pH. Pma1 internalization in yeast lacking functional V-ATPases requires the α-arrestin family protein Rim8 and the Rsp5 ubiquitin ligase (Smardon and Kane 2014). Moreover, we showed that downregulation of Pma1 through endocytosis is growth compensatory in yeast lacking functional V-ATPases, as the deletion of Rim8 or the inhibition of Rsp5 in V-ATPase (*vma*) mutants causes a strong synthetic growth defect (Smardon and Kane 2014). Interestingly, the PM levels of one of the mammalian cytosolic pH regulators, a Na^+^/H^+^ exchanger (NHE1), are also regulated by endocytosis mediated by an E3-ubiquitin ligase, Nedd4, acting in concert with β-arrestin1 (Simonin and Fuster 2010). This suggests that cross talk between organelle and PM pH regulators is conserved across species.

Rim8 was previously characterized for its role in the Rim101 signaling pathway, which senses alkaline external pH and generates a transcriptional response (Maeda 2012; Obara and Kihara 2014). High external pH is sensed by a three-protein, pH-sensing complex on the PM comprised of Rim21, Rim9, and Dfg16 (Gomez-Raja and Davis 2012; Maeda 2012). The pH signal is transduced from the pH-sensing complex via Rim8 to the downstream signal-receiving components comprised of endosomal sorting complexes required for transport proteins and a scaffold protein called Rim20, which docks the Rim101 transcription factor on the endosomal membrane (Xu *et al.* 2004; Herrador *et al.* 2010, 2015; Maeda 2012; Obara and Kihara 2014). These events culminate in the proteolytical cleavage of Rim101 and its translocation into the nucleus to modulate transcription to achieve adaptation to high external pH (Maeda 2012).

Endocytic downregulation of Pma1 in *vma* mutants was initially surprising, because Pma1 is an essential protein with a very long half-life at the PM and its levels appear to be tightly regulated (Serrano *et al.* 1986; Ferreira *et al.* 2001). However, it is well known that Pma1 activity is highly regulated post-translationally, particularly in response to glucose availability and intracellular pH (Serrano 1983; Goossens *et al.* 2000; Stratford *et al.* 2013; Mazon *et al.* 2015). This regulation is complex and incompletely understood. One regulatory pathway involves a glucose-stimulated phosphorylation of the C-terminal tail of Pma1, which has been attributed to the Ptk2 kinase and may alter the interaction of the tail with the catalytic domain (Eraso *et al.* 2006; Lecchi *et al.* 2007). However, mutations in a number of other kinases and phosphatases alter Pma1 regulation in response to glucose or cytosolic pH, but their regulatory mechanisms are not understood.

In this study, we sought to identify candidates required for Pma1 internalization to further decode signaling events in this pathway. We tested whether components of the Rim101 pathway other than Rim8 are required for Pma1internalization, and found that Rim8 plays a distinct role in Pma1 internalization. We also assessed the involvement of two phosphatases that have been implicated in both signaling through other α-arrestins and regulation of Pma1 activity, the calcium-dependent serine/threonine protein phosphatase calcineurin (CN) and a glucose-dependent serine/threonine protein phosphatase type 1 homolog Glc7. Both of these phosphatases are essential for Pma1 internalization in *vma* mutants, and we characterize their genetic and biochemical interactions with the pathway.

## Materials and Methods

### Yeast strains and media

Genotypes of yeast strains used in this study are listed in Table 1. Yeast cells were maintained in YEPD (10% yeast extract, 5% peptone, and 2% glucose buffered to pH 5, with 50 mM of potassium succinate and potassium phosphate) or in SD (fully supplemented minimal media) (0.67% yeast nitrogen base, 2% glucose, and all amino acid supplements) (Sherman 1991). *Y3656 vma2*Δ::*nat* was constructed as described (Smardon *et al.* 2014). Strain *snf1*Δ::*URA3* was obtained from Saul Honigberg (Honigberg and Lee 1998). Strain *glc7-12^ts^*::*kanMX* was obtained from Charlie Boone, University of Toronto (Li *et al.* 2011). Strain BY4741 *crz1-GFP*::*HIS3* was purchased from Invitrogen (Carlsbad, CA) and the *VMA2* gene was deleted in a single-step gene replacement to create strain *vma2*Δ::*URA3crz1-GFP* by lithium acetate transformation, as described previously (Gietz *et al.* 1992), using a PCR product amplified from the genomic DNA of a *vma2*Δ::*URA3* strain using primers VMA2(−840) and VMA2-c4 (Table 2).

**Table 1:** Yeast strains

Strain	Genotype	Source
BY4741	*MAT***a** his3Δ*1 leu2*Δ*0 met15*Δ*0 ura3*Δ*0*	Open Biosystems
BY4742	*MAT*α his3Δ*1 leu2*Δ*0 lys2*Δ*0 ura3*Δ*0*	Open Biosystems
*BY4741 vma2*Δ	*MAT***a** his3Δ*1 leu2*Δ*0 met15*Δ*0 ura3*Δ*0 vma2*Δ::*KanMx*	Open Biosystems
*Y3656 vma2*Δ	*MAT*α can1Δ:: *MFA1pr-his3-MF*α1pr-Leu2 ura3Δ*0 leu2*Δ*0 his3*Δ*1 met15*Δ*0 lys2*Δ*0 vma2*Δ::*Nat*	Kane laboratory
*BY4742 cnb1*Δ	*MAT*α his3Δ*1 leu2*Δ*0 lys2*Δ*0 ura3*Δ*0 cnb1*Δ::*Nat*	Open Biosystems
*cnb1*Δ *vma2*Δ	Spore from cross between *BY4741 vma2*Δ and *BY4742 cnb1*Δ	This study
*BY4741 reg1*Δ	*MAT***a** his3Δ*1 leu2*Δ*0 met15*Δ*0 ura3*Δ*0 reg1*Δ::*KanMx*	Open Biosystems
*BY 4741glc7-12^ts^*	*MAT***a** his3Δ*1 leu2*Δ*0 met15*Δ*0 ura3*Δ*0 glc7-12^ts^*:: *KanMx*	From Charlie Boone
*glc7-12^ts^ vma2*Δ	Spore from cross between *Y3656 vma2*Δ and *BY 4741glc7-12^ts^*	This study
*BY4741 rim20*Δ	*MAT***a** his3Δ*1 leu2*Δ*0 met15*Δ*0 ura3*Δ*0 rim20*Δ::*KanMx*	Open Biosystems
*BY4741 rim21*Δ	*MAT***a** his3Δ*1 leu2*Δ*0 met15*Δ*0 ura3*Δ*0 rim21*Δ::*KanMx*	Open Biosystems
*vma2*Δ *rim20*Δ	Spore from cross between *Y3656 vma2*Δ and *BY 4741 rim20*Δ	This study
*vma2*Δ *rim21*Δ	Spore from cross between *Y3656 vma2*Δ and *BY 4741 rim21*Δ	This study
*BY4741 rim8*Δ	*MAT***a** his3Δ*1 leu2*Δ*0 met15*Δ*0 ura3*Δ*0 rim8*Δ::*KanMx*	Open Biosystems
*vma2*Δ *rim8*Δ	Spore from cross between *Y3656 vma2*Δ and *BY 4741 rim8*Δ	Kane laboratory
*BY4741 ptk2*Δ::*hygB*	*MAT***a** his3Δ*1 leu2*Δ*0 met15*Δ*0 ura3*Δ*0 ptk2*Δ::*HygB*	This study
*BY4741 ptk2*Δ::*kan*	*MAT***a** his3Δ*1 leu2*Δ*0 met15*Δ*0 ura3*Δ*0 ptk2*Δ::*kanMx*	Open Biosystems
*vma2*Δ *ptk2*Δ *glc7-12^ts^*	Spore from cross between *glc7-12^ts^ vma2*Δ and *BY4741 ptk2*Δ::*hygB*	Open Biosystems
BY4741 *crz1*Δ	*MAT***a** his3Δ*1 leu2*Δ*0 met15*Δ*0 ura3*Δ*0 crz1*Δ::*kan*	Open Biosystems
*vma2*Δ *crz1*Δ	Spore from cross between *Y3656 vma2*Δ and *BY 4741 crz1*Δ	This study
*SH snf1*Δ	W303 *snf1*Δ::*URA3*	From Saul Honigberg
*BY 4741 crz1-GFP*	*MAT***a** his3Δ*1 leu2*Δ*0 met15*Δ*0 ura3*Δ*0 crz1-GFP*	Invitrogen
*BY 4741 crz1-GFP vma2*Δ	*MAT***a** his3Δ*1 leu2*Δ*0 met15*Δ*0 ura3*Δ*0 vma2*Δ::*URA3 crz1-GFP*	This study
*BY4741 Pma1-007*::*KanMx*	*MAT***a** his3Δ*1 leu2*Δ*0 met15*Δ*0 ura3*Δ*0 Pma1-007*::*KanMx*	Open Biosystems
*BY4741 Pma1-007*::*HygB*	*MAT***a** his3Δ*1 leu2*Δ*0 met15*Δ*0 ura3*Δ*0 Pma1-007*::*HygB*	Kane laboratory
BY4741 *Rim8 P506A*-3HA	*MAT***a** his3Δ*1 leu2*Δ*0 met15*Δ*0 ura3*Δ*0 Rim8 P506A-3HA-KanMx*	This study
*vma2*Δ *Rim8 P506A*-3HA	Spore from cross between *Y3656 vma2*Δ*/* pRS316 vma2-URA3 and BY4741 *Rim8 P506A*-3HA	This study
*vma2*Δ *Pma1-007*	Spore from cross between *vma2*Δ *Rim8 P506A*-3HA/pRS316 vma2-URA3 and *BY4741 Pma1-007*::*HygB*	This study
*Pma1-007 Rim8 P506A*-3HA	Spore from cross between *vma2*Δ *Rim8 P506A*-3HA/pRS316 vma2-URA3 and *BY4741 Pma1-007*::*HygB*	This study
*vma2*Δ *Rim8P506A*-3HA *Pma1-007*	Spore from cross between *vma2*Δ *Rim8 P506A*-3HA/pRS316 vma2-URA3 and *BY4741 Pma1-007*::*HygB*	This study
BY4741 *Rim8*-3HA::*KanMx*	*MAT***a** his3Δ*1 leu2*Δ*0 met15*Δ*0 ura3*Δ*0 Rim8*-3HA::*KanMx*	Kane laboratory
*BY4741 rcy1*Δ	*MAT***a** his3Δ*1 leu2*Δ*0 met15*Δ*0 ura3*Δ*0 rcy1*Δ::*KanMx*	Open Biosystems

**Table 2:** Primers used in this study

Primer name	Sequence (5′–3′)
VMA2(−840)	GAATCGGCTAGAGATTACAAGCTCA
VMA2-c4	GATGTTCTTCGAGACCGGGTTGG
Rim8:451URAfor	TTC AAA GAT ATG GTA AAT GTG GAA AAG CTA AAG AGA CTG AGG AAT GTA ACT GGT TAC TGA GAG TGC ACC ACGCT
Rim8:end URArev	ATA GTC ATC ACA AGG GGG AGG ATC GCT TTC TAA CTG TTG TAG TCT TTT TTG TTC AAG CAG TTT TTT AGT TTG CTG GCC
P506A.For	TTTATCATTTGGCGAATACTTTGCAACTGGAACATCGTCACTATCATA
P506A.Rev	GTCAGTGTACCGTCGGAAGACAGACAAGAACTTGAACAAAAAAGAC
Rim8-F2	TTAATTAACCCGGGGATCCGATAGTCATCACAAGGGGGAGGATC
Rim8-R1	GTTTAAACGAGCTCGAATTCAAAGTGCAGGTAACAAGTCATATACTCG
Rim8 553	GAAGCAAGATGCGAACGTGAG

To generate double and triple mutants, congenic haploid strains harboring single gene deletions were crossed, and the resulting diploids were sporulated for ∼3–5 days as described (Kassir and Simchen 1991). Tetrads were dissected, and the genotypes of the individual spores were confirmed via the selectable markers. The double mutants *vma2*Δ *cnb1*Δ, *vma2*Δ *crz1*Δ, *vma2*Δ *rim8*Δ, *vma2*Δ *rim20*Δ, *vma2*Δ *rim21*Δ, *vma2*Δ *rimP506A-3HA*, and *vma2*Δ *glc7-12^ts^* were created by sporulation and tetrad dissection after crossing strains BY4741 *vma2*Δ::*kanMx* and BY4742 *cnb1*Δ::*nat*, *Y3656 vma2*Δ::*nat* and BY4741 *crz1*::*kanMx*, *Y3656 vma2*Δ::*nat* and BY4741 *rim8*Δ::kanMx, *Y3656 vma2*Δ::*nat* and BY4741*rim20*Δ::*kanMx*, *Y3656 vma2*Δ::*nat* and BY4741*rim21*Δ::*kanMx*, *Y3656 vma2*Δ::*nat* and BY4741*rim8 P506A-3HA*::*kanMx*, and *Y3656 vma2*Δ::*nat and glc712^ts^*::*kanMx*, respectively. Triple mutant *vma2*Δ *glc7-12^ts^ptk2*Δ was created by tetrad dissection by crossing strains *vma2*Δ *glc7-12^ts^* and *BY4741ptk2*Δ::*hygB*. Double mutants *vma2*Δ *pma1-007* and *rimP506A-3HA pma1-007*, and triple mutant *vma2*Δ *rimP506A-3HA pma1-007*, were obtained by tetrad dissection as described above by crossing strains *vma2*Δ *rimP506A-3HA*, carrying the *VMA2* gene on plasmid pRS316, and *BY4741 pma1-007*::*hygB*. Growth of spores of different genotypes were compared by growing liquid cultures of each strain to log phase, then spotting 10-fold serial dilutions onto the relevant plates. The plates were incubated at the appropriate temperature for at least 3 days.

To make the BY4741*rim8* P506A-3HA::*kanMx* mutant, we first PCR amplified the *URA3* gene from the pRS316 plasmid, flanked by ∼50-bp sequences upstream and downstream of Rim8 amino acids 451–539, using primers Rim8:451URAFor and Rim8:endURARev (Table 2). This PCR product was introduced into the BY4741 Rim8-3HA-kanMx strain (see below), replacing the sequence for amino acids 451–539 with *URA3* to construct the BY4741 Rim8450-URA3-Rim8STOP-3HA::kanMx strain. The Rim8 P506A mutation was first introduced into a plasmid-borne copy of *RIM8* using primers P506AFor and P506ARev (Table 2) with a quick-change site-directed mutagenesis kit (Stratagene, La Jolla, CA) and sequenced. A fragment containing the Rim8 P506A mutation was then PCR amplified using primers Rim8553 and Rim8R1 (Table 2) and transformed into BY4741 Rim8450-URA3-Rim8STOP-3HA::kanMx. Transformants were plated on plates containing 5-fluoroorotic acid (5-FOA) to select for replacement of *URA3*. Incorporation of the Rim8 P506A mutation was confirmed by sequencing.

To tag Rim8 at its C-terminus with a 3HA (hemagglutinin) tag, the 3HA-kanMx6 cassette from plasmid pFA6a-3HA-kanMX6 (Longtine *et al.* 1998) was amplified using primers Rim8 F2 and Rim8 R1 (Table 2). The cassette was introduced into the BY4741 wild-type strain, transformants resistant to G418 (kan) were selected, and in-frame incorporation of the tag was confirmed by sequencing.

### Fluorescence microscopy

We used indirect immunofluorescence to observe Pma1 localization. Cells were grown to log phase and then fixed and permeabilized as described (Roberts *et al.* 1991). The fixed and permeabilized cells were washed with 1% SDS for 1 min, then immobilized on glass slides coated with 1 mg/ml polylysine (Roberts *et al.* 1991) and incubated overnight at room temperature with a 1:200 dilution of anti-Pma1 antibody (mouse monoclonal 40B7; Novus Biologicals). The next day, cells were gently washed with ice cold PBS-BSA (5 mg/ml bovine serum albumin in phosphate-buffered saline) and incubated for 1 hr at room temperature with a 1:300 dilution of Alexa-fluor 488-conjugated goat anti-mouse secondary antibody from Invitrogen (Martinez-Munoz and Kane 2008; Smardon and Kane 2014). The cells were washed finally with PBS-BSA and layered with mounting media before covering with a cover slip. Cells were visualized using differential interference contrast, and fluorescein isothiocyanate filters on a Zeiss ([Carl Zeiss], Thornwood, NY) Imager Z1 fluorescence microscope (Smardon and Kane 2014) attached to a Hamamatsu CCD camera. Images were obtained using Zeiss Axiovision software.

To observe the Pma1 localization in *glc7-12^ts^* and *glc7-12^ts^vma2*Δ cells, cells were initially grown to log phase at the permissive 25°, followed by pelleting and resuspension of ∼10 OD_600_ of cells in YPD pH 5 prewarmed to the nonpermissive 37°. The cells were incubated at the nonpermissive temperature of 37° for 2 hr before fixation and visualization. To inhibit CN in a *vma2*Δ mutant, cells were grown to midlog phase (0.6 OD_600_) then diluted to 0.2 OD_600_ and divided into equal volumes. The diluted *vma2*Δ cells were treated for 2, 4, and 6 hr with 10 μg/ml of the CN-specific inhibitor FK-506 (Tacrolimus) purchased from AG Scientific. The DMSO solvent for the FK-506 was applied in parallel as a vehicle treatment. At each time point, DMSO and the FK-506-treated cells were collected, fixed, and permeabilized for Pma1 indirect immunofluorescence. To localize Pma1 after acute inhibition of V-ATPases, we treated the wild-type cells and the *pma1-007* cells grown to the midlog phase with 2 μM of the V-ATPase inhibitor concanamycin A for 30 min, followed by fixation and visualization of Pma1 indirect immunofluorescence as described above.

Wild-type and *vma2*Δ cells expressing Crz1-GFP were grown to midlog phase in minimal media. To mark the cell nucleus, cells were incubated with 2.5 mg/ml of DAPI (4’,6-diamidino-2-phenylindole, dihydrochloride) stain at 30° for 30 min. Then cells were washed once with minimal media and resuspended in minimal media. Crz1-GFP was visualized with a GFP filter set and DNA was visualized with a DAPI filter set.

### FM 4-64 uptake assay

As a measure of bulk endocytosis, we observed uptake of the lipophilic dye N-(3-triethylammoniumpropyl)-4-(6-(4-(diethylamino) phenyl) hexatrienyl pyridinium dibromide (FM 4-64) (Molecular Probes, Eugene, OR/Invitrogen). To assess the effect of PP1 inhibition, wild-type, *vma2*Δ, *glc7-12^ts^*, and *glc7-12^ts^vma2*Δ cells were allowed to grow to midlog phase in YEPD pH 5. For each strain, three separate aliquots of cell suspension (equivalent to 1 OD_600_ unit of cells) were pelleted and resuspended in ice-cold YEPD pH 5 containing 20 μM FM 4-64. To initially label only the PMs, cells were pulse labeled on ice with continuous shaking for 20 min (Vida and Emr 1995), washed with ice-cold fully supplemented minimal media to reduce background fluorescence, and imaged using a Texas Red filter. The remaining two tubes from each mutant were washed twice with YEPD pH 5 prewarmed to either 25° (permissive for *glc7-12^ts^*) or 37° (nonpermissive for *glc7-12^ts^*). After pelleting to remove excess dye, the pellets were resuspended in YEPD pH 5 and chased for 2 hr either at 25 or 37°, then washed with fully supplemented minimal media and imaged as above. To check the effects of CN inhibition, *vma2*Δ cells were grown to midlog phase and distributed across seven tubes (cell volumes equivalent to 1 OD_600_ units in each). Pelleted cells were resuspended in 1 ml ice-cold YEPD pH 5 containing FM 4-64 as described above. After a 20 min pulse labeling, one tube of cells was washed and imaged as described above, and the other tubes were washed twice with room temperature YEPD pH 5 and resuspended in 1 ml of YEPD pH 5. FK-506 was added (final concentration 10 μg/ml) to three tubes and DMSO was added to the other three. Samples were chased at 30° for 2, 4, and 6 hr, then washed and imaged as described above.

### Quantification and statistical analysis of the Pma1 indirect immunofluorescence

PM fluorescence of the Pma1 in all the experiments was quantified using National Institutes of Health (NIH) Image J version 2.0.0-rc-54/1.51f as described (Smardon and Kane 2014). We adapted the cell fluorescence quantification methods described (Gavet and Pines 2010; McCloy *et al.* 2014) with slight modifications. An outline was drawn using one of the Image J shape tools around the outer edge of the yeast cell and area, integrated density, and mean gray value were measured. Then using the same shape, similar measurements were taken in the background adjacent to the cell. The “Total Cell Fluorescence” (TCF) was calculated using the formula “Integrated density – (area × mean gray value of the background).” Next, using a shape tool, an outline was drawn inside the cell just below the PM, again measuring the same parameters as above, and “Fluorescence Internal to the PM” (FI) of the cell was calculated using the same formula. The “PM Fluorescence Intensity” (PMFI) of the cell was calculated by subtracting the FI from the TCF. Finally, the PMFI of the cell was divided by the integrated density of the cell to get “Normalized PM fluorescence intensity” (NPMFI). In every biological replicate, 52 cells from at least six different fields were quantified for each cell type. All the well-focused cells in a field were measured. The NPMFI between different cell types in an experiment were compared using bar charts, and a paired Student’s *t*-test was performed using GraphPad to determine if the difference between mean NPMFI among different cell types was significant. *P*-value ≤ 0.05 was considered significant.

### Immunoprecipitation and immunoblotting

To test the effect of inhibition of PP1 on the ubiquitination of Pma1, the *glc7-12^ts^* and the *glc7-12^ts^vma2*Δ cells were initially grown to midlog phase (0.6 OD_600_) in YPD pH 5 at 25°. Cell suspension equivalent to 60 OD_600_ units was pelleted and resuspended in medium prewarmed to 37° for 2 hr. Wild-type and *vma2*Δ cells were used as negative and positive controls, respectively, and a *glc7-12^ts^* suspension maintained at 25° was also included. Equal cell numbers (determined from OD_600_) were pelleted and frozen at −80° until immunoprecipitation. To test the effect of CN on Pma1 ubiquitination, midlog (0.6 OD_600_) phase cultures were diluted to 0.2 OD_600_ and treated in separate flasks with either DMSO or FK-506 (10 μg/ml) for 2, 4, and 6 hr at 30°. Wild-type cells and the untreated *vma2*Δ cells were used as negative and positive controls, respectively, and all samples were pelleted and frozen as described above.

Immunoprecipitations were performed as described in Smardon and Kane (2014), with some modifications. The frozen cell pellets were resuspended in 300 μl ice-cold lysis buffer (50 mM Tris-HCL pH 7.5, 100 mM NaCl, and 0.1 mM EDTA) with protease, phosphatase, and deubiquitinase inhibitors (1 mM PMSF, 1 μg/ml leupeptin, 5 μg/ml aprotinin, 2 μg/ml pepstatin, 10 mM NaF, 1 mM Na_3_VO_4_, and 25 mM N-ethylmaleimide). The cells were lysed by agitation with an equal volume of acid-washed glass beads (300 μl) at 4°. Next, 1% Triton-X 100, 1% sodium deoxycholate, and 0.1% SDS were added to the lysate, and the mixture was incubated on ice for 30 min. Lysates were then diluted with 1 ml of ice-cold lysis buffer (with all the inhibitors) to decrease the concentration of detergent, followed by centrifugation for 5 min to remove the insoluble material. The supernatant was incubated with 50 μl Protein A Sepharose beads for 20 min on ice to preabsorb proteins binding nonspecifically [Protein A Sepharose CL-4B powder (GE Healthcare) (17 mg/sample) was swollen overnight in PBS-BSA and washed in PBS-BSA for three times before use]. After centrifugation, the supernatant was incubated overnight at 4° with 7 μl of anti-mouse monoclonal antibody against Pma1 (40B7) purchased from Novus Biologicals. The next day, 60 μl of 50% (v/v) suspension of protein A Sepharose CL-4B was added to the samples and incubated at 4° for 2 hr. Then the immunoprecipitate was washed three times with the ice-cold lysis buffer and solubilized in cracking buffer (50 mM Tris-HCL, pH 6.8, 8 M urea, 5% SDS, and 5% β-mercaptoethanol) at 55° for 10 min. The immunoprecipitates were analyzed by electrophoresis using an 8% SDS-polyacrylamide gel and western blotting. Identical volumes from all the samples were loaded into separate wells of the gel. Total Pma1 in each sample was compared by probing with 1:5000 of anti-rabbit polyclonal Ab against Pma1 (a generous gift from Amy Chang), and the ubiquitinated portion of Pma1 was visualized and compared by probing with 1:250 of anti-mouse (P4D1) monoclonal IgG against ubiquitin (Santa Cruz Biotechnology). Secondary antibodies conjugated with alkaline phosphatase (Promega, Madison, WI) were used to visualize protein bands using an AP conjugate substrate kit (Bio-Rad, Hercules, CA). Three independent experiments were performed and the band intensities were quantified using NIH Image J version 2.0.0-rc-54/1.51f as described in Smardon and Kane (2014).

Phosphorylated and total Snf1 were visualized in the presence and absence of glucose, as described in Orlova *et al.* (2008) with some modifications. Whole-cell lysates were prepared as described in Orlova *et al.* (2008) for western blotting. To detect phosphorylated Snf1, blots were probed with a 1:1000 dilution of phospho-AMPKα (Thr172) antibody (Cell Signaling Technology) in TBST (20 mM Tris-HCl, pH 7.5, 500 mM NaCl, and 0.1% Tween 20) incubated overnight at 4°. Total endogenous Snf1 was detected by probing with monoclonal anti-poly-histidine antibody (H1029) (Sigma [Sigma Chemical], St. Louis, MO) at a concentration of 1:3000 in 1% milk incubated overnight at 4°. Protein was detected and quantified as described (Smardon and Kane 2014).

### Data availability

All reagents and supporting data are available upon request.

## Results

### Pma1 internalization in V-ATPase mutants is independent of the Rim101 pathway

**Figure 1:**
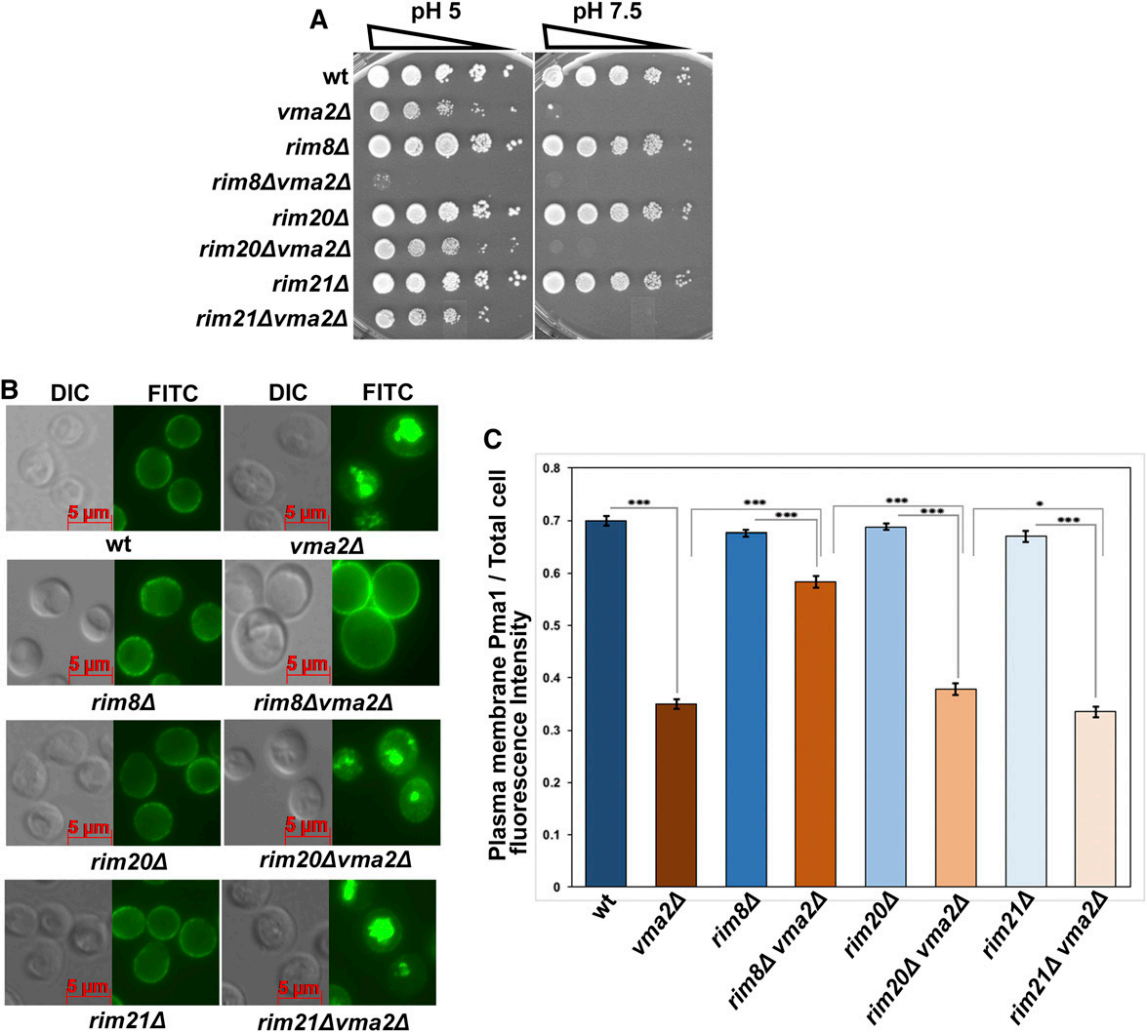
The internalization of Pma1 in the yeast lacking functional V-ATPase is independent of the components required for the alkaline pH-sensing Rim101 signaling pathway. (A) Dilution growth assay comparing the growth of *rim20*Δ*vma2*Δ, *rim21*Δ*vma2*Δ, and *rim8*Δ*vma2*Δ double mutants with their corresponding single mutants and wild-type (wt) cells. The upstream pH sensor component Rim21 and the downstream scaffold protein rim20 are the required components for Rim101 signaling during external alkaline pH stress, and α-arrestin Rim8 transduces the signal between pH sensor and receiving complexes. We made the *rim20*Δ*vma2*Δ, *rim21*Δ*vma2*Δ, and *rim8*Δ*vma2*Δ double mutants as described in the *Materials and Methods* using tetrad dissection. (B) Anti-Pma1 indirect immunofluorescent image showing the localization of Pma1 in *rim20*Δ*vma2*Δ, *rim21*Δ*vma2*Δ, and *rim8*Δ*vma2*Δ double mutants, their corresponding single mutants, and wt cells. A representative of three independent experiments is shown. (C) Bar graph comparing the plasma membrane Pma1 fluorescence intensity as a fraction of total cell fluorescence intensity (arbitrary units) in different rim101 pathway and *vma* double mutants. Pma1 fluorescence intensity quantified for 52 cells from each mutant as described in the *Materials and Methods*. The *P*-value ≤ 0.05 is considered significant. In the bar graph, * *P* < 0.05 and *** *P* < 0.0005. *P* > 0.05 is considered nonsignificant and nonsignificant differences are not indicated in the bar graph. The error bars indicate ± SEM.

Rim8 is a required component of the Rim101 pathway, so we tested whether other essential components of the Rim101 signaling pathway are required for the Pma1 internalization pathway. We crossed deletion mutants of the upstream pH sensor component Rim21, which has been shown to interact with Rim8 (Herrador *et al.* 2015) or the downstream scaffold protein Rim20, with a *vma2*∆ mutant and obtained double-mutant strains by tetrad dissection. Loss of proteins required for Pma1 internalization further compromise the growth of yeast *vma* mutants (Smardon and Kane 2014), so we compared the growth of the *rim21*Δ*vma2*Δ and the *rim20*Δ *vma2*Δ double mutants to that of wild-type cells and the three single mutants using a dilution growth assay (Figure 1A). The *vma* mutants have a distinctive growth phenotype characterized by very poor growth at elevated pH (pH 7.5) and optimal growth at pH 5. The *rim20*Δ *vma2*Δ and *rim21*Δ*vma2*Δ double mutants grew similarly to the *vma2*Δ cells. In contrast, the *rim8*∆*vma2*∆ double mutant grows extremely poorly, even at pH 5, consistent with previous results (Smardon and Kane 2014).

Next, we tested whether the *vma* mutants require Rim21 and Rim20 for Pma1 internalization. Using anti-Pma1 indirect immunofluorescence in the fixed cells, we localized Pma1 in the *rim21*Δ*vma2*Δ and *rim20*Δ *vma2*Δ double mutants and their corresponding single mutants *rim21*Δ and *rim20*Δ (Figure 1B). As expected, the *rim21*Δ and *rim20*Δ single mutants retained Pma1 on the PM, similarly to the wild-type cells, whereas the *rim21*Δ*vma2*Δ and *rim20*Δ *vma2*Δ double mutants internalized Pma1 similarly to the *vma2*Δ cells. Quantification of the PM Pma1 fluorescence intensity in these mutants (Figure 1C) showed that the *rim20*Δ *vma2*Δ double mutant had slightly higher Pma1 levels at the PM compared to the *rim21*Δ*vma2*Δ mutant. However, in contrast to *rim8*∆*vma2*∆, neither the *rim20*∆*vma2*∆ nor the *rim21*∆*vma2*∆ double mutant had significantly different levels of PM fluorescence from the single *vma2*∆ mutant (Figure 1, B and C). These results show that Rim101 pathway components Rim21 and Rim20 are not essential for Pma1 internalization in *vma* mutants, and suggest that the Rim101 pathway is not required. Although the α-arrestin Rim8 is a common component in both the pathways, it appears to perform distinct and independent functions depending on the signal it receives.

### Yeast lacking V-ATPase function require PP1 for growth and endocytic downregulation of Pma1

The activity of Pma1 is post-translationally regulated by phosphorylation and dephosphorylation in response to glucose availability (Eraso and Portillo 1994; Estrada *et al.* 1996; Lecchi *et al.* 2007; Mazon *et al.* 2015). The Reg1 protein is one of several regulatory subunits of a highly conserved, type 1 serine/threonine protein phosphatase, PP1, which directs the activity of a single catalytic subunit encoded by yeast *GLC7* (Tu and Carlson 1995; Sanz *et al.* 2000). The Glc7-Reg1 complex is believed to reduce Pma1 activity during glucose starvation by dephosphorylating Ser 899 on the Pma1 C-terminus (Williams-Hart *et al.* 2002; Mazon *et al.* 2015), and *reg1*Δ cells have higher Pma1 activity even during glucose starvation (Young *et al.* 2010). Furthermore, Reg1 interacts directly with the α-arrestin Rod1/Art4 and may promote Rsp5-dependent, ubiquitin-mediated endocytosis of the PM lactate transporter Jen1 (Becuwe *et al.* 2012). Considering the role of Glc7 in both the regulation of Pma1 activity and the endocytosis of other PM transporters, we hypothesized that PP1 could be involved in Pma1 endocytosis as well.

**Figure 2:**
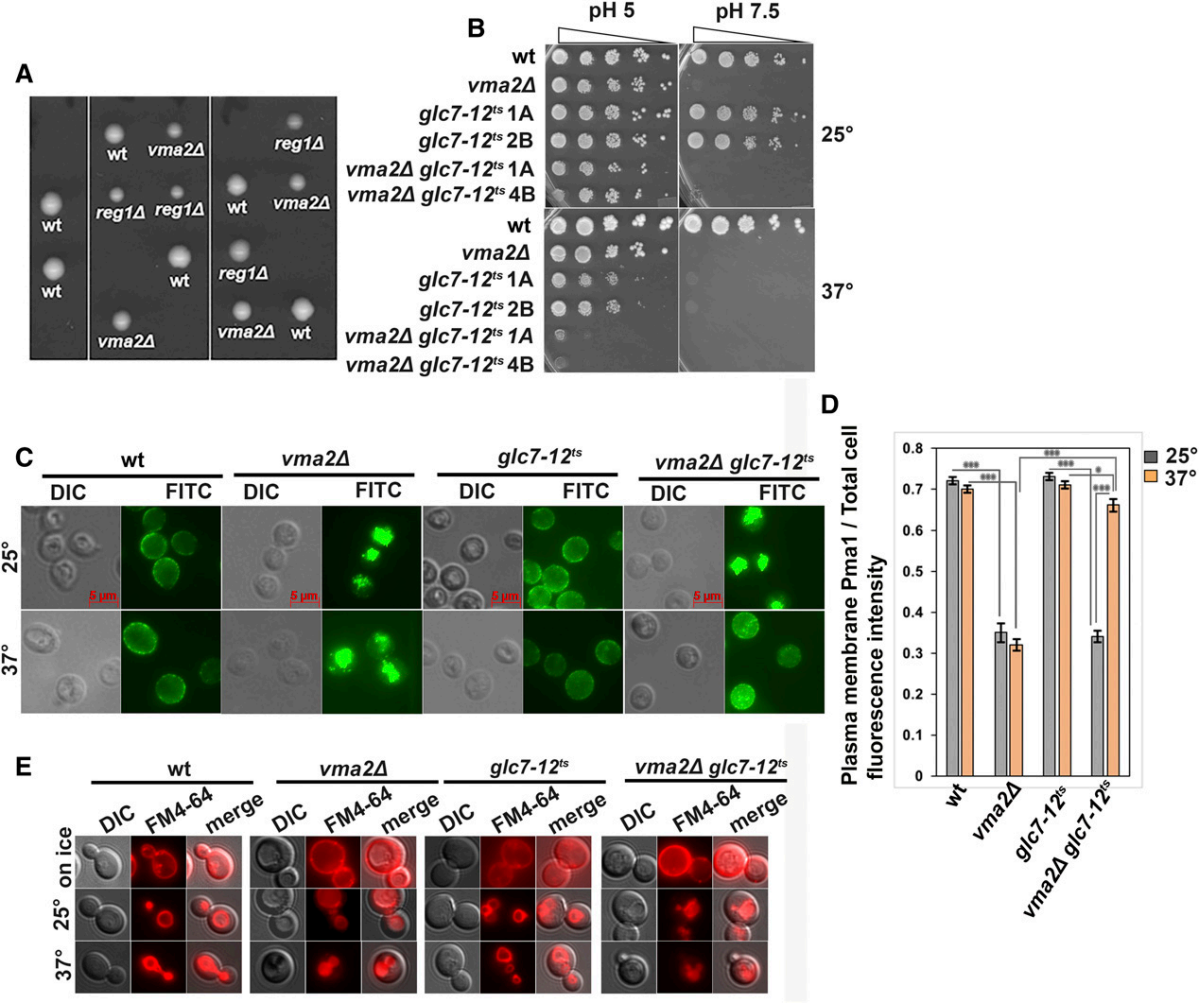
Protein phosphatase type 1 (PP1) is required for the growth of the yeast lacking functional V-ATPases and is required for the internalization of Pma1. (A) The tetrads obtained by crossing a PP1 regulatory subunit mutant, BY4741 *reg1*Δ::*kan*, with *vma* mutant y3536*vma2*Δ::*nat* cells. (B) Dilution growth assay comparing the growth of a *glc7-12^ts^ vma2*Δ double mutant (two double mutants from two different tetrads are shown) with the corresponding *glc7-12^ts^* single mutant (two single mutants from two different tetrads are shown), *vma2*Δ cells, and wild-type (wt) cells at permissive temperature of 25° to that of at the nonpermissive temperature of 37°. The double mutant was obtained by tetrad dissection at 25° by crossing a temperature-sensitive PP1 catalytic subunit mutant, *glc7-12^ts^*:: *kan*, with a *vma* mutant, y3536 *vma2*Δ::*nat*. The *vma* phenotype is verified by checking the growth at YPD pH 7.5 as the *vma* mutant phenotype is conditionally lethal at higher pH. (C) Anti-Pma1 indirect immunofluorescence (see *Materials and Methods*) image of fixed cells comparing the localization of Pma1 in the *glc7-12^ts^vma2*Δ double mutant with the corresponding *glc7-12^ts^* single mutant, *vma2*Δ cells, and the wt cells. The *glc7-12^ts^vma2*Δ double-mutant cells and the glc7-12^ts^ single-mutant cells were initially grown to the log phase in YPD pH 5 at the permissive temperature of 25°; then, to inhibit the PP1 catalytic subunit Glc7, these mutants were shifted to the nonpermissive temperature of 37° for 2 hr. A representative of three independent experiments is shown. (D) Bar graph showing the plasma membrane (PM) Pma1 fluorescence intensity as a fraction of total cell fluorescence intensity (arbitrary units) in different cells in (C). From each strain, 52 cells were quantified for the fluorescent intensity of Pma1 at the PM as a measure of the amount of Pma1 retaining at the PM, as described in the *Materials and Methods*. The *P*-value ≤ 0.05 is considered significant. In the bar graph, * *P* < 0.05 and *** *P* < 0.0005. *P* > 0.05 is considered nonsignificant and nonsignificant differences are not indicated in the bar graph. The error bars indicate ± SEM. (E) Micrograph showing the uptake of FM 4-64 dye in wt, *vma2*Δ, *glc7-12^ts^*, and *vma2*Δ *glc7-12^ts^* cells. Cells were initially labeled on ice to mark only the PMs and immediately imaged using a Texas Red filter. Later, cells were chased for 2 hr at either 25 or 37°, as described in the *Materials and Methods*, and imaged using Texas a Red filter to monitor the uptake of FM 4-64 from the PM to the vacuole.

To test this hypothesis, we first attempted to make a *reg1*Δ*vma2*Δ double mutant through tetrad dissection. However, we obtained no viable double-mutant spores, as shown by the five tetrads in Figure 2A, indicating that the two mutations may be synthetically lethal. The PP1 catalytic subunit Glc7 is an essential protein in yeast, so we utilized a temperature-sensitive mutant *glc7-12^ts^* (MacKelvie *et al.* 1995) and generated *vma2*Δ *glc7-12^ts^* double mutants by tetrad dissection at the permissive temperature of 25°. Figure 2B shows that at the permissive temperature, the *vma2*Δ *glc7-12^ts^* double mutants grow similarly to the *vma2*Δ cells. However, at the nonpermissive temperature of 37°, the *vma2*Δ *glc7-12^ts^* mutants grew very poorly even at pH 5, corroborating the synthetic lethal phenotype of the *reg1*Δ*vma2*Δ mutant. This negative synthetic growth phenotype suggested that Glc7-Reg1 could be a candidate in the Pma1 internalization pathway. Surprisingly, the temperature sensitivity of the *glc7-12^ts^* single-mutant spores is suppressed at pH 5. We previously observed that the temperature sensitivity of the *rsp5-1* single mutant is also suppressed at pH 5, while the *rsp5-1vma2*∆ mutant grew very poorly (Smardon and Kane 2014).

We examined Pma1 localization in the *glc7-12^ts^* single and *vma2*Δ *glc7-12^ts^* double mutant at both temperatures (Figure 2C). The *glc7-12^ts^* mutant maintains wild-type levels of Pma1 at the PM at both temperatures (Figure 2D). At 25°, internalization of Pma1 in the *vma2*Δ *glc7-12^ts^* double mutant was similar to that in the *vma2*Δ cells. Remarkably, after a 2 hr shift to 37°, the *vma2*Δ *glc7-12^ts^* mutant retained wild-type levels of Pma1 at the PM, unlike *vma2*Δ cells. This result shows that loss of PP1 activity in *vma* mutants inhibits Pma1 endocytic downregulation. However, the inhibitory effect of PP1 on the Pma1 endocytosis in *vma2*Δ cells could be due to a more general effect of PP1 inhibiting bulk endocytosis from the PM. To check this, we assayed wild-type cells and *vma2*Δ, *glc7-12^ts^* and *vma2*Δ *glc7-12^ts^* mutants for the uptake of a bulk endocytosis marker, a lipophilic dye, FM 4-64. Uptake of FM 4-64 from the PM and its delivery to the vacuole is dependent on endocytosis, and it has been used as a vacuolar marker (Vida and Emr 1995). Figure 2E shows that on ice, FM 4-64 labeled only PMs in all the cell types. However, after chasing for 2 hr at either the permissive temperature of 25° or the nonpermissive 37°, the FM 4-64 internalized and labeled vacuolar membranes in all the cell types shown. This result shows that PP1’s effect on Pma1 internalization is not due to an overall inhibition of endocytosis. Instead, these results indicate a specific requirement for PP1 in the Pma1 internalization pathway in *vma* mutants.

Serine 899 (S899) in the Pma1 cytosolic C-terminus was previously identified as the target of Glc7-mediated dephosphorylation under glucose starvation (Mazon *et al.* 2015). We hypothesized that if Glc7-mediated dephosphorylation of S899 of Pma1 is critical for its endocytic downregulation, we should able to rescue the poor growth of the *vma2*Δ *glc7-12^ts^* double mutant at the nonpermissive temperature by deleting the kinase that phosphorylates Pma1 S899. Ptk2 kinase was recently shown to be solely responsible for S899 phosphorylation (Mazon *et al.* 2015), so we constructed a *glc7-12^ts^vma2*∆*ptk2*∆ triple mutant. If dephosphorylation of S899 were required for Pma1 endocytosis, we expected that the *glc7-12^ts^vma2*Δ*ptk2*Δ triple mutant would grow better than the *vma2*Δ *glc7-12^ts^* double mutant at the nonpermissive temperature. However, the dilution growth assay showed that there was no significant growth enhancement of the *glc7-12^ts^vma2*Δ *ptk2*Δ triple mutant relative to the *vma2*Δ *glc7-12^ts^* double mutant at 37° (Supplemental Material, Figure S1). This result argues that the phosphorylation state of Pma1 S899 is not a critical determinant of Pma1 endocytosis, but that Glc7 may target some other site on Pma1 or another protein in the Pma1 internalization pathway.

The Glc7-Reg1 complex has been associated with glucose signaling and plays a significant role in glucose repression (Tu and Carlson 1995; Sanz *et al.* 2000). During glucose starvation, the yeast homolog of AMP-activated protein kinase, Snf1, is activated by phosphorylation and modulates transcription to support the use of alternate carbon sources (McCartney *et al.* 2016). Upon glucose readdition to glucose-starved cells, the Glc7-Reg1 complex dephosphorylates Snf1 and inactivates it (Sanz *et al.* 2000). *vma* mutants are subject to multiple stresses, so we asked whether Snf1 is activated in *vma* mutants even without glucose starvation; if so, the Reg1 requirement in *vma* mutants might be directed toward limiting Snf1 activity. We observed the activation state of Snf1 in wild-type, *vma2*Δ, *snf1*Δ, and *reg1*Δ whole-cell lysates by probing western blots with antibodies recognizing total and phosphorylated Snf1. The western blot shown in Figure S2 shows that wild-type and *vma2*Δ cells have comparable levels of total Snf1, a protein that is phosphorylated to similar levels in the absence of glucose. In contrast, *reg1*Δ cells have higher levels of phosphorylated Snf1 even in the presence of glucose and levels increase further under glucose starvation, characteristic of Snf1 hyperactivation. Thus, Snf1 is not hyperactive in *vma* mutants under normal growth conditions and is unlikely to be the critical Reg1/Glc7 target in the Pma1 internalization pathway. Instead, Reg1 may direct Glc7 to another critical component.

### PP1 acts upstream of Pma1 ubiquitination in the endocytic downregulation pathway

**Figure 3:**
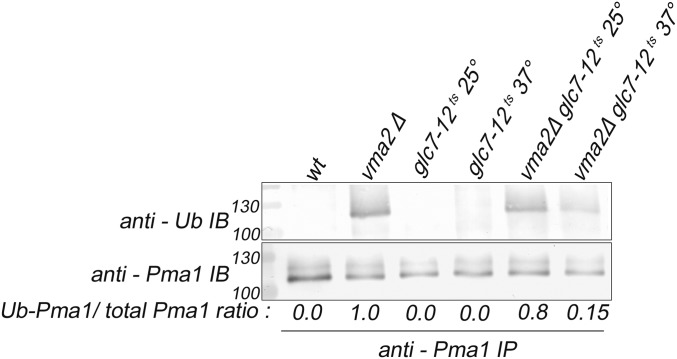
Protein phosphatase type 1 (PP1) is required for the ubiquitination of Pma1 in the Pma1 endocytic downregulation pathway in yeast lacking a functional V-ATPase; the PP1 catalytic subunit *glc7-12^ts^* single mutant and the *glc7-12^ts^vma2*Δ double mutant cells are grown to the log phase at the permissive temperature of 25°, and then they are shifted to the nonpermissive temperature of 37° for 2 hr. The Pma1 is immunoprecipitated from the shown mutants as described in the *Materials and Methods*. The immunoprecipitates were probed with either anti-Pma1 antibody to observe the total Pma1 using western blotting or anti-ubiquitin antibody to observe the ubiquitinated portion of the Pma1. The amount of Pma1 ubiquitinated in each cell type is expressed as the ratio of the ubiquitinated portion of the Pma1 to that of the total Pma1. The intensity of the Pma1 bands was measured using National Institutes of Health Image J. The representative of three different experiments is shown. wt, wild-type.

Ubiquitination of Pma1 is a critical step in its internalization (Smardon and Kane 2014), so we examined whether Pma1 ubiquitination is impaired in a PP1 mutant. We immunoprecipitated Pma1 from wild-type and mutant cells and probed the immunoprecipitated protein with anti-Pma1 antibody to assess total Pma1 levels, and with anti-ubiquitin antibody to assess the extent of Pma1 ubiquitination (Figure 3). We previously demonstrated that wild-type cells contain little, if any, ubiquitinated Pma1, but that *vma2*∆ mutants contain both lower levels of total Pma1 and increased Pma1 ubiquitination (Smardon and Kane 2014). We did not detect any ubiquitinated Pma1 in wild-type cells or the *glc7-12^ts^* cells at either temperature, consistent with the PM localization of Pma1 (the anti-Pma1 antibody frequently shows multiple bands on immunoblots, as shown in Figure 3, but this does not necessarily indicate ubiquitination.) In the *vma2*Δ cells, total levels of Pma1 were somewhat lower than in wild-type cells, and higher levels of ubiquitination were present, as expected. At 25°, the *vma2*Δ *glc7-12^ts^* double mutant shows increased Pma1 ubiquitination like in the *vma2*∆ strain, and the ratio of the ubiquitin signal to the Pma1 signal resembled that of the *vma2*∆ strain. However, ubiquitinated Pma1 is undetectable when the double mutant is shifted to 37° for 2 hr. This result demonstrates that ubiquitination of Pma1 in *vma* mutants requires PP1, and places PP1 activity upstream of the critical step of Rim8- and Rsp5-dependent ubiquitination of Pma1.

### Endocytic downregulation of Pma1 requires CN

CN is a highly conserved, Ser/Thr protein phosphatase activated by Ca^2+^/calmodulin binding. In yeast, stresses such as high concentrations of certain ions, high external pH, high temperature, and high osmolarity result in elevation of cytosolic [Ca^2+^], activating CN (Ruiz *et al.* 2008; Ariño 2010; Cyert and Philpott 2013; Alvaro *et al.* 2014; Thewes 2014; Guiney *et al.* 2015). Yeast CN is a heterodimer with the catalytic subunit encoded by either of the redundant *CNA1* and *CNA2* genes, and the regulatory subunit encoded by the *CNB1* gene. Both subunits are required for CN function. CN exerts its effects primarily through the activation of the transcription factor Crz1. Under normal conditions, Crz1 is phosphorylated and inactive in the cytosol. However, when dephosphorylated by CN, it translocates into the nucleus, binds to DNA, and modulates transcription (Yoshimoto *et al.* 2002; Cyert 2003).

Although wild-type yeast cells do not require CN for growth under normal conditions, CN has been reported to be essential in *vma* mutants (Garrett-Engele *et al.* 1995; Sambade *et al.* 2005; Zhao *et al.* 2013). The *vma* mutants have higher basal cytosolic [Ca^2+^] because the vacuolar H^+^/Ca^2+^ exchanger Vcx1 cannot operate efficiently in the absence of the vacuolar H^+^ gradient (Ohsumi and Anraku 1983; Forster and Kane 2000). Although defective Ca^2+^ homeostasis could account in part for the synthetic lethality of *vma* and CN mutations, we hypothesized that CN might also be required for Pma1 internalization in *vma* mutants. Interestingly, CN was also reported to control Pma1 activity (Hemenway *et al.* 1995), and CN regulates endocytosis of some PM transporters by directly modulating the activity of α-arrestins independent of the transcription factor Crz1 (O’Donnell *et al.* 2013; Alvaro *et al.* 2014, 2016).

**Figure 4:**
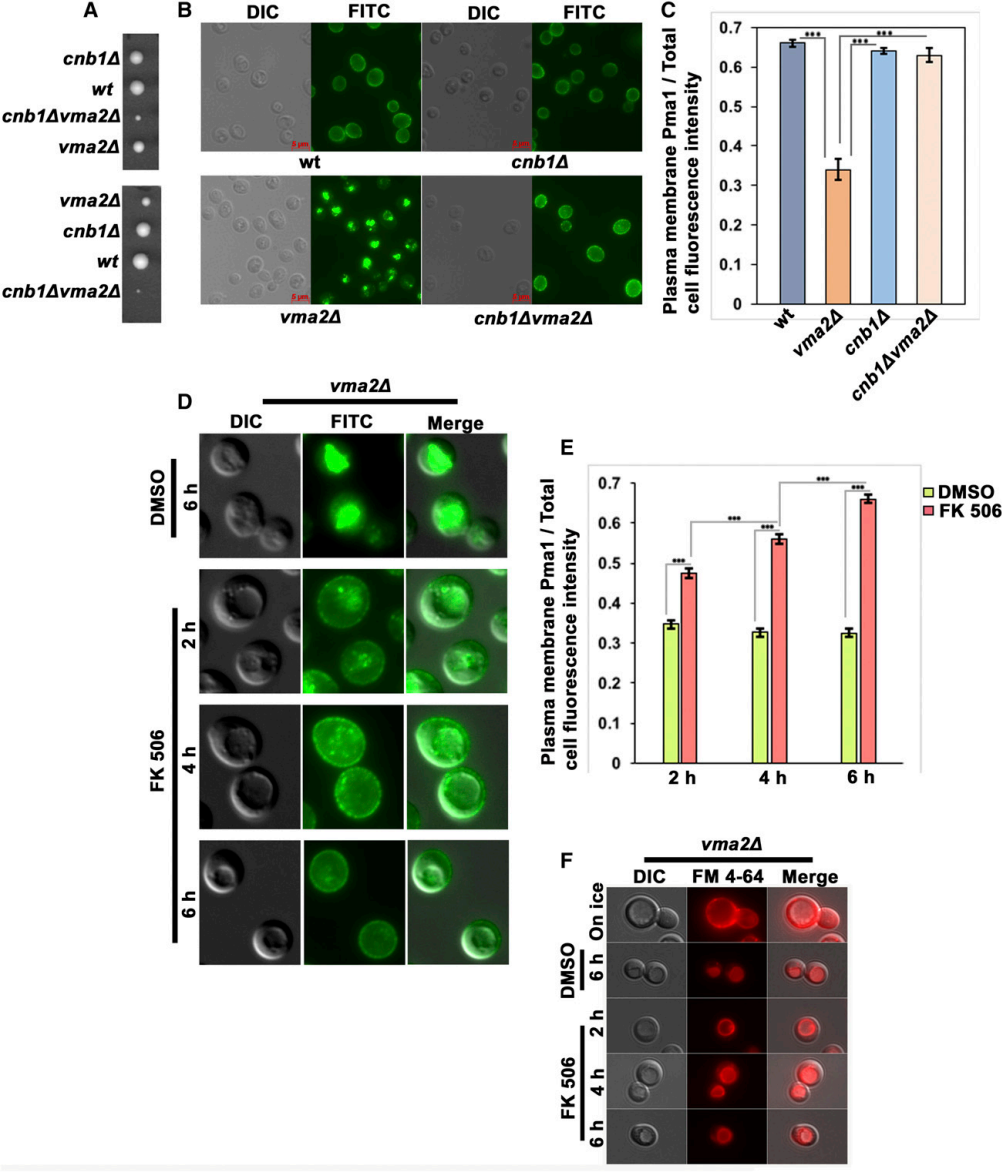
Calcineurin is required for the internalization of Pma1 in the yeast cells lacking functional V-ATPases. (A) Two tetrads obtained from the cross of a calcineurin mutant, BY 4742 *cnb1*Δ::*nat*, with a *vma* mutant, BY 4741*vma2*Δ::*kan*. The *cnb1*Δ*vma2*Δ double mutant grows very poorly compared to the single mutants. (B) Anti-Pma1 indirect immunofluorescence from the spores in a tetrad shown in (A). The cells were allowed to grow in YEP dextrose medium pH 5 to the log phase, and were then fixed as described in the *Materials and Methods* to monitor the localization of Pma1. (C) Bar graph showing the plasma membrane Pma1 fluorescence intensity as a fraction of total cell fluorescence intensity (arbitrary units) in different cells in (B). From each strain, 52 cells were quantified for the fluorescent intensity of Pma1 at the plasma membrane as a measure of the amount of Pma1 retaining at the plasma membrane, as described in the *Materials and Methods*. The *P*-value ≤ 0.05 was considered significant. In the bar graph, *** *P* < 0.0005. *P* > 0.05 was considered nonsignificant and nonsignificant differences were not indicated in the bar graph. The error bars indicate ± SEM. (D) The *vma2*Δ cells were treated with either DMSO or with 10 μg/ml of a calcineurin inhibitor, FK506. The cells were collected 2, 4, and 6 hr after treatment and fixed to observe the Pma1 localization using anti-Pma1 indirect immunofluorescence. In the DMSO-treated cells, only the 6 hr time point is shown. A representative of three different experiments is shown. (E) Bar graph showing the plasma membrane Pma1 fluorescence intensity as a fraction of total cell fluorescence intensity (arbitrary units) from cells in (D). From each condition, 52 cells were quantified for the fluorescent intensity of Pma1 at the plasma membrane as a measure of the amount of Pma1 retaining at the plasma membrane, as described in the *Materials and Methods*. The *P*-value ≤ 0.05 was considered significant. In the bar graph, *** *P* < 0.0005. *P* > 0.05 was considered nonsignificant and nonsignificant differences are not indicated in the bar graph. The error bars indicate ± SEM. (F) Micrograph showing the uptake of FM 4-64 dye in *vma2*Δ cells treated with either DMSO or the calcineurin inhibitor FK506. Cells were initially labeled on ice to mark only the plasma membranes and immediately imaged using a Texas Red filter. Later, cells were chased at 30° for 2, 4, and 6 hr in the presence of either DMSO or the calcineurin inhibitor FK506, as described in the *Materials and Methods*, and imaged using a Texas Red filter to monitor the uptake of FM 4-64 from the plasma membrane to the vacuole. wt, wild-type.

To assess whether CN has a role in the Pma1 endocytic downregulation, we made a *CNB1* and V-ATPase double mutant, *cnb1*Δ*vma2*Δ, through tetrad dissection. Although deletion of CN in *vma* mutants was reported to be synthetically lethal (Garrett-Engele *et al.* 1995), we observed extremely poor growing *cnb1*Δ*vma2*Δ double mutants at low temperature on YPD pH 5 plates (Figure 4A). In Figure 4, B and C, we determined the localization of Pma1 in each of the four spores of one of the tetrads in Figure 4A. In the *cnb1*Δ mutant that lacks CN activity, Pma1 localizes at the PM similarly to the wild-type cells. As expected, the *vma2*Δ cells constitutively internalize a portion of Pma1. In contrast, the *cnb1*Δ*vma2*Δ double-mutant cells retained Pma1 at the PM. To confirm the requirement of CN for Pma1 internalization, we treated *vma2*Δ cells, with the CN-specific inhibitor FK506 (Breuder *et al.* 1994) for 2, 4, and 6 hr (Figure 4, D and E), and checked the localization of Pma1 at each time point. After 2 hr of FK506 treatment, *vma2*Δ cells still had a substantial amount of Pma1 inside the vacuole, but had more Pma1 at the PM than the vehicle (DMSO)-treated control. At 4 hr, PM localization of Pma1 was increased, and by 6 hr of FK506 treatment Pma1 was entirely at the PM. This result shows that inhibiting CN inhibits Pma1 internalization in *vma* mutants, consistent with the *cnb1*Δ*vma2*Δ double-mutant phenotype in Figure 4, B and C.

CN’s inhibitory effect on Pma1 internalization could be due to the inhibition of bulk endocytosis from the PM. As with PP1, we assayed the uptake of FM 4-64 dye in *vma2*Δ cells treated with FK506 (Figure 4F). As in Figure 2, *vma2*Δ cells pulse labeled on ice with FM 4-64 exhibited only PM fluorescence, but when chased at 30° for 2, 4, and 6 hr in the presence of FK506 the FM 4-64 internalized and labeled vacuolar membranes, indicating that CN inhibition does not inhibit endocytosis. These results show that CN is required for Pma1 internalization in *vma* mutants.

**Figure 5:**
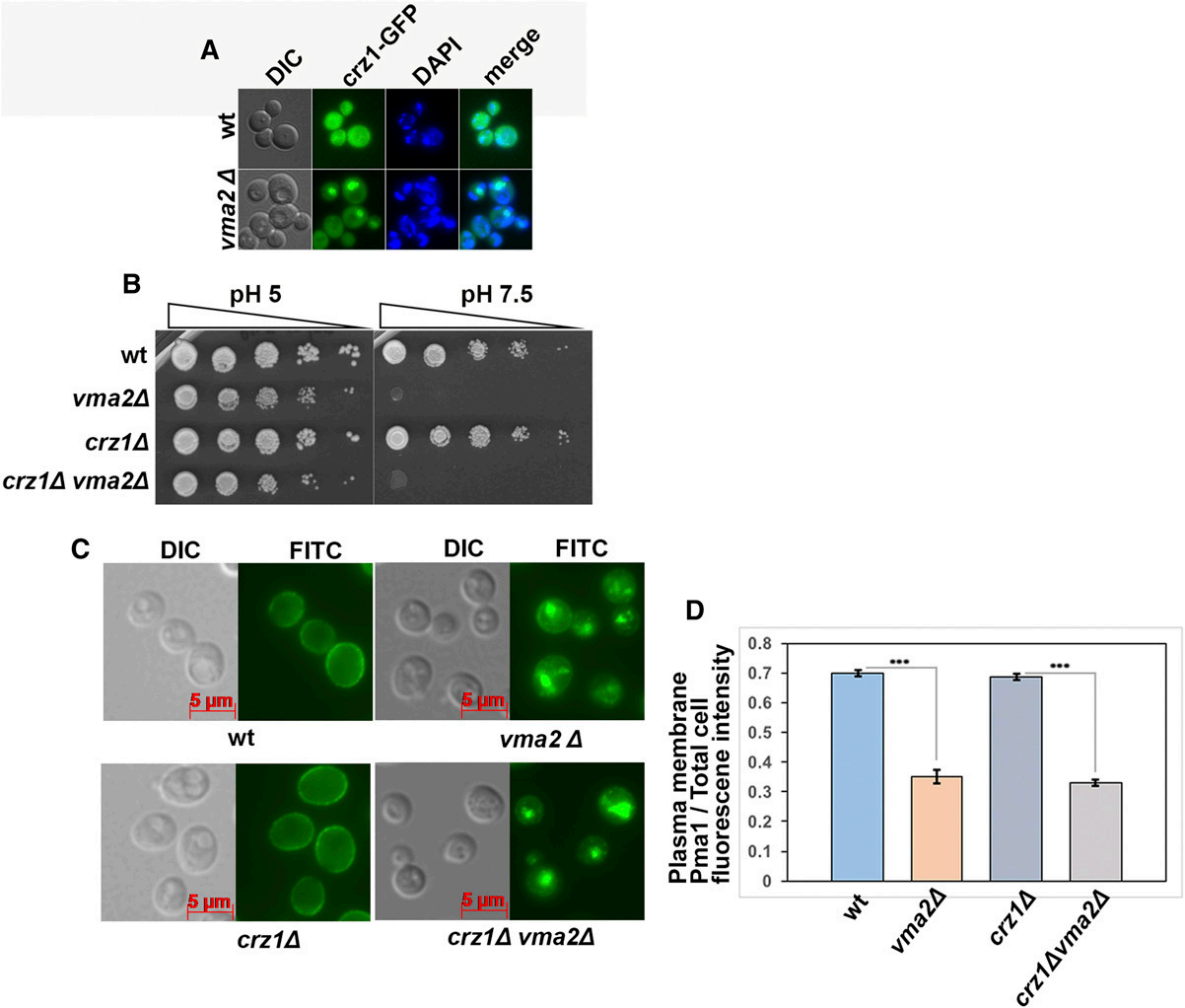
Endocytic downregulation of the Pma1 in the yeast lacking a functional V-ATPase is independent of the calcineurin-dependent transcription factor Crz1. (A) Calcineurin-dependent transcription factor Crz1 is tagged with GFP in wild-type (wt) and *vma2*Δ cells, and the localization of Crz1-GFP is monitored using a fluorescent microscope. To identify the nucleus, the cells were stained with DAPI. (B) Dilution growth assay comparing the growth of the *crz1*Δ*vma2*Δ double mutant with the corresponding single mutants. The *crz1*Δ*vma2*Δ double mutant is made as described in the *Materials and Methods*. (C) Anti-Pma1 indirect immunofluorescence image comparing the localization of Pma1 in the *crz1*Δ*vma2*Δ double mutant with the corresponding single mutants and wt cells. The cells were fixed for immunofluorescence as described in the *Materials and Methods*. A representative from three independent experiments is shown. (D) Bar graph showing the plasma membrane Pma1 fluorescence intensity as a fraction of total cell fluorescence intensity (arbitrary units) from cells in (C). From each strain, 52 cells were quantified for the fluorescence intensity of Pma1 at the plasma membrane as a measure of the amount of Pma1 retaining at the plasma membrane, as described in the *Materials and Methods*. The *P*-value ≤ 0.05 is considered significant. In the bar graph, *** *P* < 0.0005. *P* > 0.05 is considered nonsignificant and nonsignificant differences are not indicated in the bar graph. The error bars indicate ± SEM. wt, wild-type.

CN could be driving the internalization of Pma1 by modulating transcription through the activation of the CN-dependent transcription factor Crz1 or directly targeting some critical component in the pathway. We examined the localization of Crz1 tagged with GFP in wild-type and *vma* mutant cells. Inactive Crz1 is a cytosolic protein, and in wild-type cells Crz1-GFP localizes diffusely in the cytosol (Figure 5A). However, in 30% of *vma2*Δ cells, Crz1-GFP is concentrated in the nucleus (only 7% of wild-type cells have nuclear Crz1-GFP). This provides further support for constitutive CN activation in *vma* mutants and suggests that CN-dependent transcription could affect Pma1 localization. If this were true, we would predict that *vma* mutants require the Crz1 protein for normal growth. We made the *crz1*Δ*vma2*Δ double mutant through tetrad dissection and compared its growth with corresponding single mutants using a dilution growth assay (Figure 5B). The *crz1*Δ single mutant has no growth defects under the conditions checked, and the *crz1*Δ*vma2*Δ double mutant grows comparably to the *vma2*Δ mutant, in contrast to the severe growth defect of the *cnb1*∆*vma2*∆ mutant. In *crz1*∆ *vma2*∆ double mutants, Pma1 internalization was similar to that in *vma2*Δ cells (Figure 5, C and D). This result indicates that in the absence of V-ATPase activity, CN is driving Pma1 internalization not by modulating transcription through Crz1, but by directly acting on another critical component in the pathway.

### CN acts upstream of Rim8/Rsp5-dependent ubiquitination of Pma1

**Figure 6:**
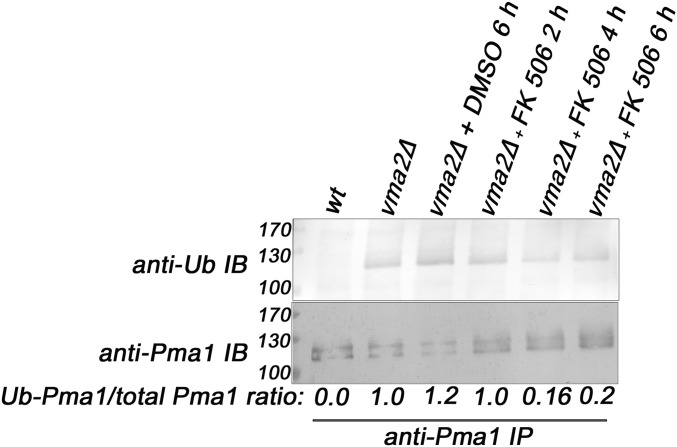
Calcineurin is required for the ubiquitination of Pma1 in the Pma1 endocytic downregulation pathway in the yeast lacking functional V-ATPases. The V-ATPase mutant *vma2*Δ cells were treated with either DMSO for 6 hr or with the calcineurin inhibitor FK506 for 2, 4, and 6 hr, then the Pma1 was immunoprecipitated from these cells, as described in the *Materials and Methods*. The immunoprecipitates were probed with either anti-Pma1 antibody to observe the total Pma1 using western blotting or anti-ubiquitin (Ub) antibody to observe the ubiquitinated portion of the Pma1. The amount of Pma1 ubiquitinated in each sample is expressed as the ratio of the ubiquitinated portion of the Pma1 to that of the total Pma1. The Intensity of the Pma1 bands was measured using National Institutes of Health Image J. A representative from three independent experiments is shown. IP, immunoprecipitation; wt, wild-type.

To further probe the role of CN in the Pma1 internalization, we examined the effect of loss of CN activity on Pma1 ubiquitination. We immunoprecipitated Pma1 from wild-type cells, and from *vma2*Δ cells treated with either DMSO or the CN inhibitor FK506, for 2, 4, and 6 hr (Figure 6). Immunoprecipitated proteins were probed with anti-Pma1 and anti-ubiquitin antibodies as described above. The wild-type cells had no ubiquitinated Pma1 and the *vma2*Δ cells, untreated or treated with DMSO, contain ubiquitinated Pma1. However, after 4 and 6 hr of FK506 treatment, the level of ubiquitinated Pma1 had declined, as had the ratio of the ubiquitin signal to the total Pma1 signal. This is consistent with the retention of PM Pma1 at these time points in Figure 4, D and E. Although the *vma2*Δ cells treated with FK506 for 2 hr appeared to have more Pma1 at the PM than the DMSO-treated cells in Figure 4, D and E, the level of ubiquitinated Pma1 was similar to that of the DMSO-treated *vma2*Δ χελλσ. These results suggest that CN acts upstream of Pma1 ubiquitination and endocytosis in the *vma* mutants.

### Does reduced PM Pma1 compensate for loss of V-ATPase activity?

Yeast cells cannot survive without Pma1 activity, but yeast *vma* mutants have roughly half the level of Pma1 protein and activity in the PM (Martinez-Munoz and Kane 2008; Tarsio *et al.* 2011; Smardon and Kane 2014). As shown above and in previous work, mutations that block internalization of Pma1 cause severe synthetic growth phenotypes when combined with *vma* mutations. These results indicate that if *vma* mutants endocytose all the Pma1 they will die, and if they do not internalize some of the Pma1 they will still die. The intriguing question is how the *vma* mutants are tackling this challenge. In other words, how do they sense the level of Pma1 internalization that provides the maximum growth advantage?

**Figure 7:**
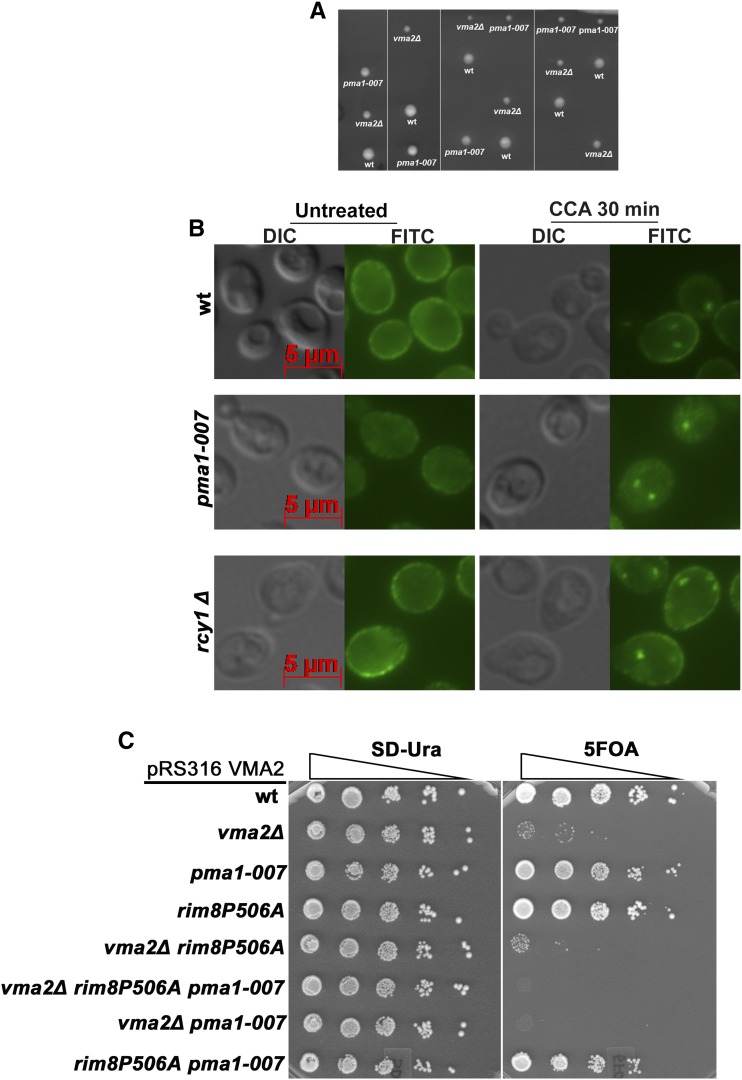
Yeast cells lacking functional V-ATPases continue to signal the downregulation of Pma1 through endocytosis, even when the Pma1 expression is reduced by half. (A) The tetrads obtained by crossing the *Pma1-007* strain, which expresses 50% less Pma1, with the *vma2*Δ cells to isolate the *pma1-007vma2*Δ double mutant through tetrad dissection. In the tetrads obtained, double-mutant spores failed to grow. (B) Anti-Pma1 indirect immunofluorescence image showing the localization of Pma1 in the wild-type (wt), *pma1-007*, and *rcy1*∆ cells either untreated or treated with 2 μM of the V-ATPase inhibitor concanamycin A. (C) Dilution growth assay comparing the growth of triple mutant *vma2*∆ *rim8P506A Pma1007* with the corresponding single and double mutants. The triple and double mutants in this figure are made as described in the *Materials and Methods*.

The *pma1-007* strain has a small deletion in the *PMA1* promoter region and, as a result, expresses only ∼50% of the wild-type Pma1 level (Porat *et al.* 2005). Because this represents a reduction in PM Pma1 that is very similar to the reduction seen in vma2∆ mutants (Smardon and Kane 2014; and Figure 1C, Figure 2D, Figure 4E, and Figure 5C), we hypothesized that *pma1-007* mutation might rescue growth of the *vma* mutants without the need for Pma1 endocytosis. No double-mutant spores were obtained after sporulation of a diploid heterozygous for the *vma2*∆ and *pma1-007* mutations, indicating that *vma2*∆ and *pma1-007* mutations may be synthetically lethal (Figure 7A). To investigate the source of the synthetic lethality, we treated the *pma1-007* mutant with the V-ATPase inhibitor concanamycin A and observed Pma1 localization (Figure 7B). The untreated *pma1-007* mutant has lower levels of PM Pma1, as expected. After 30 min of concanamycin A treatment, both wild-type and *pma1-007* cells contain internal puncta corresponding to internalized Pma1. This result indicates that inhibition of the V-ATPase in *pma1-007* cells triggers the Pma1 internalization despite already reduced levels of Pma1 on the PM. Therefore, it appears that *vma* mutants cannot sense the amount of Pma1 at the PM and continue to signal for Pma1 downregulation, likely resulting in too little PM Pma1 activity to support viability.

We hypothesized that the prevention of Pma1 internalization in the *pma1-007vma2*Δ double mutant by deleting *RIM8* might rescue growth. We crossed a *vma2*∆*rim8*∆ mutant carrying wild-type *VMA2* on a pRS316 (CEN, *URA*) plasmid (pRS316-*VMA2*) with the *pma1-007* mutant, then sporulated the resulting diploid. We obtained triple mutant *vma2*Δ *rim8*Δ *pma1-007* spores carrying the *VMA2* plasmid. However, when we tried to evict the plasmid on plates containing 5-FOA, the triple mutants did not grow (data not shown). Because a *rim8*∆ mutant might have pleiotropic effects, we also used a Rim8 PY motif mutant, which cannot bind to the Rsp5 ubiquitin ligase. We generated a *vma2*Δ *rim8P506A-3HA pma1-007* triple mutant spore bearing pRS316-*VMA2*. As shown in Figure 7C, the triple mutant cannot evict the plasmid and grow on 5-FOA medium, indicating that blocking Pma1 endocytosis did not rescue growth of the *vma2*Δ *rim8*Δ *pma1-007* triple mutant. The double mutant *vma2*Δ *rim8P506A-3HA* grew very poorly, like the *vma2*∆*rim8*∆ mutant (Figure 1A). Double mutant *vma2*Δ *pma1-007* also failed to grow on 5-FOA plates, corroborating the result in Figure 7A. Taken together, these results indicate that simple downregulation of PM Pma1 activity may not fully account for the compensatory effects of Pma1 internalization in *vma* mutants. Instead, it may also be important to populate endosomal compartments with Pma1, before its final delivery and degradation within the vacuole.

If this is the case, we might expect that when V-ATPase activity is lost, internalized Pma1 gains access to internal compartments that it does not generally occupy. To begin to address this, we examined Pma1 localization in an *rcy1*∆ mutant, which blocks PM proteins in an early endosome after endocytosis (Wiederkehr *et al.* 2000), and also traps several transporters that constitutively cycle through early endosomes and back to the PM (MacDonald and Piper 2017). As described previously (Wiederkehr *et al.* 2000), Pma1 does not recycle and thus remains completely at the PM in an *rcy1*∆ mutant. However, after a 30 min treatment with concanamycin A, Pma1 is observed in puncta in the *rcy1*∆ mutant that are very similar to those seen in wild-type cells in Figure 7C. This suggests that internalized Pma1 reaches early endosomes, and could potentially contribute to their acidification, although we cannot directly measure endosomal pH at present.

## Discussion

**Figure 8:**
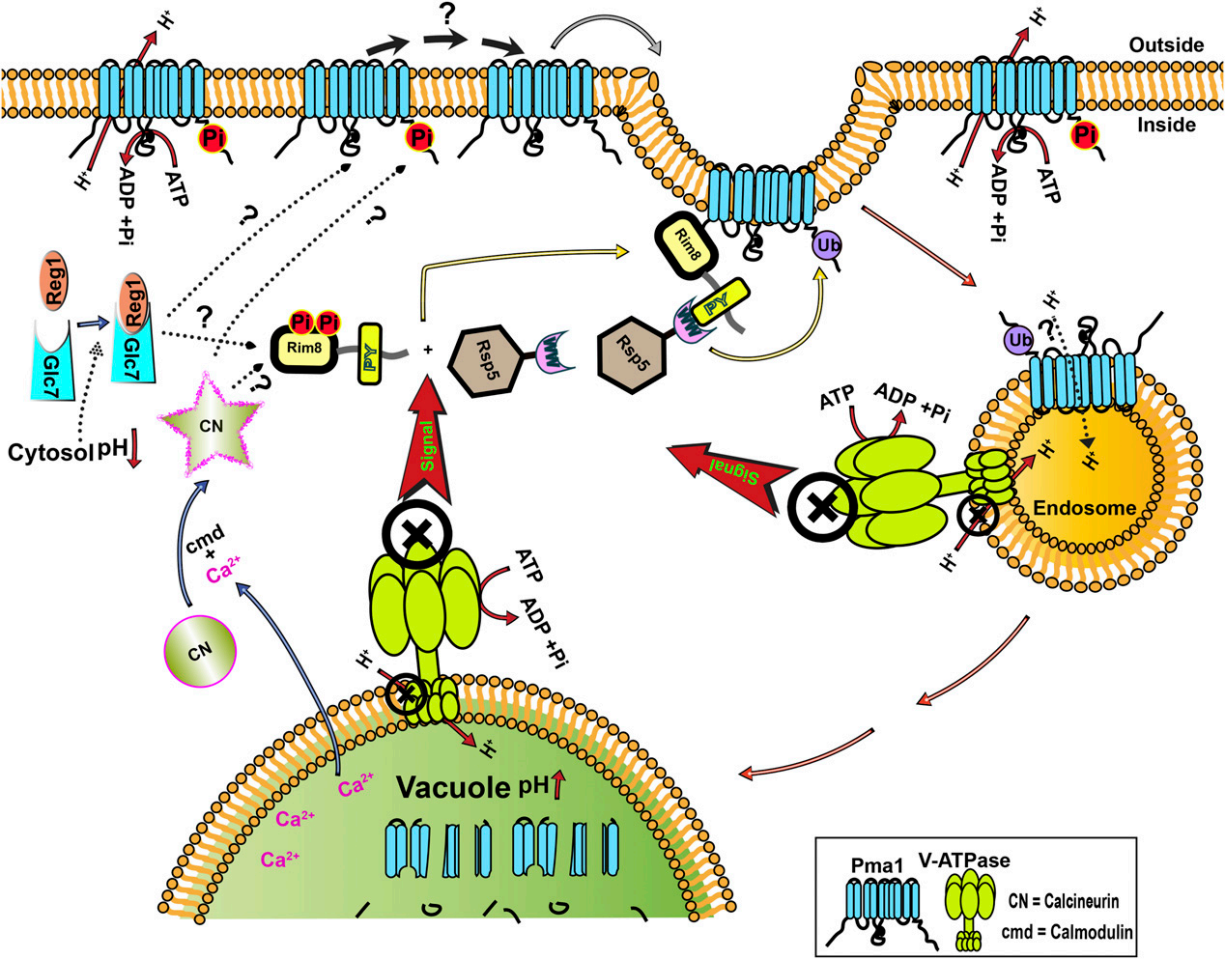
Schematic showing a working model for Pma1 downregulation in yeast lacking functional V-ATPases. Lack of V-ATPases at acidic organelles such as vacuoles and endosomes results in increased organelle pH and decreased cytosolic pH. Moreover, lack of V-ATPase signals ubiquitination of ∼50% of Pma1 at the plasma membrane by E3-Ubiquitin ligase Rsp5, aided by the PY motif containing α-arrestin Rim8, leading to the internalization of Pma1 through endocytosis to finally degrade in the vacuolar lumen. Two phosphatases Glc7 (PP1) and calcineurin (CN) are required for the ubiquitination and downregulation of Pma1 through endocytosis. While the targets of these phosphatases are yet to be determined, the schematic shows potential targets of the phosphatases in dotted arrows with question marks. cmd, calmodulin.

A scheme for ubiquitination and endocytosis of Pma1 is shown in Figure 8. Consistent with models developed for other α-arrestins (Lin *et al.* 2008; Hatakeyama *et al.* 2010; Becuwe *et al.* 2012; Hovsepian *et al.* 2017), we envision Rim8 as detecting a loss of V-ATPase activity and acting as an adaptor to bring Rsp5 to the PM, where it ubiquitinates a subpopulation of Pma1 at the PM, targeting this population for endocytosis and degradation. In this work, we add two new players to this pathway, conserved protein phosphatases CN and Glc7/Reg1, and provide further evidence that endocytosis of Pma1 is essential when organelle acidification is compromised, suggesting that it represents a critical compensatory pathway required for rebalancing pH homeostasis. Finally, we present evidence that endocytosis of Pma1 may play a role beyond partial clearance of the proton pump from the membrane to downregulate its activity.

How cells recognize loss of V-ATPase activity and signal this loss to Rim8, and ultimately to Pma1, is an important question. Loss of balance between V-ATPase and Pma1 activity has been invoked as a factor in yeast replicative aging (Henderson *et al.* 2014), and the significance of intercompartmental contributions to overall pH balance is highlighted by recent work (Gottschling and Nystrom 2017). This pH-balancing act between compartments can occur on both short and long timescales. Although *vma* mutants experience a chronic loss of V-ATPase activity, we have previously demonstrated that V-ATPase inhibition with concanamycin A elicits ubiquitination and endocytosis of Pma1 in < 30 min (Smardon and Kane 2014). This suggests that the response can be rapid, consistent with that observed in other pathways involving α-arrestins (Hatakeyama *et al.* 2010; Becuwe *et al.* 2012), as well as constitutive in the *vma* mutants. The experiments described have eliminated some candidate pathways for cross talk between organellar V-ATPases and PM Pma1, while suggesting other pathways. Specifically, although Rim8 plays an essential role in Pma1 ubiquitination and endocytosis, it appears to act independently of the RIM ambient pH response pathway, since neither upstream sensor Rim21 nor downstream effector Rim20 of the RIM pathway (Penalva *et al.* 2008; Maeda 2012) are required for Pma1 endocytosis. This result suggests that Rim8 is “moonlighting” in the Pma1 endocytic pathway. The early steps in the RIM pathway are geared toward sensing extracellular pH (Penalva *et al.* 2008), but in the Pma1 internalization pathway it is likely that Rim8 detects changes in organelle or cytosolic pH directly or indirectly. We have not eliminated the possibility that Rim8 is a direct pH sensor, but the requirements for protein phosphatases CN and Glc7/Reg1 in the Pma1 endocytic pathway suggest other possible mechanisms through which cells might recognize a loss of V-ATPase activity.

The requirement for CN suggests that elevated cytosolic Ca^2+^ might help signal a loss of V-ATPase activity. We previously measured cytosolic Ca^2+^ responses in the *vma* mutants and in the presence of concanamycin A (Forster and Kane 2000). In fungi, the vacuole is a major Ca^2+^ store, and one of the most important uptake mechanisms is a Ca^2+^/H^+^ exchanger that exploits the V-ATPase-generated proton gradient (Miseta *et al.* 1999). However, CN is involved in recognizing other stresses as well, ranging from high salt to pH stress (Cyert and Philpott 2013), so we cannot assume that elevated cytosolic Ca^2+^ alone serves as a surrogate signal for loss of vacuolar acidification. Interestingly, CN control of Pma1 activity has been described previously (Withee *et al.* 1998). Expression of a constitutively active form of CN in wild-type cells decreased Pma1 activity (Withee *et al.* 1998). Plants lack CN, but plant Pma1 activity was decreased when a constitutively active form of yeast CN was heterologously expressed in tomato (Marín-Manzano *et al.* 2004). In these settings, localization of Pma1 was not determined, but the results are consistent with CN downregulating Pma1 activity. In contrast, the requirement for Glc7/Reg1 evokes the well-established links between glucose availability and pH sensing (Goossens *et al.* 2000; Dechant *et al.* 2010). The Glc7/Reg1 complex is involved in recovery from starvation and is the major phosphatase involved in downregulating Snf1 activity (Tu and Carlson 1995). We have shown that Snf1 activity is similar between wild-type and *vma2*∆ cells (Figure S2). However, Glc7/Reg1 has also been associated with pH control. Specifically, Pma1 activity and cytosolic pH are higher in a *reg1*∆ mutant under glucose-limited conditions (Young *et al.* 2010), suggesting that the mutant fails to adjust Pma1 activity during glucose deprivation and consistent with a role for Glc7/Reg1 in Pma1 downregulation. Glucose deprivation lowers the cytosolic pH and raises the vacuolar pH, mimicking the conditions in *vma* mutants, where vacuolar pH is higher and cytosolic pH is lower even in the presence of glucose (Martinez-Munoz and Kane 2008; Tarsio *et al.* 2011). It is possible that loss of V-ATPase activity could transmit a signal similar to glucose deprivation via cytosolic pH changes.

Placement of CN and Glc7 activity in the scheme for Rim8-mediated signaling of Pma1 endocytosis (Figure 8) is difficult because the targets of these phosphatases remain unclear. We demonstrate here that Glc7 and CN are required for Pma1 ubiquitination in response to loss of V-ATPase activity (Figure 3 and Figure 6), placing them upstream of the Rsp5-mediated ubiquitination step in Figure 8. The α-arrestins themselves are often highly phosphorylated proteins that, in some cases, are also ubiquitinated by Rsp5 (Lin *et al.* 2008; MacGurn *et al.* 2011; Merhi and Andre 2012; Herrador *et al.* 2013; Hovsepian *et al.* 2017). Phosphorylation patterns on the α-arrestins change in response to signals for transporter downregulation, and their activation often requires dephosphorylation. For example, CN-mediated dephosphorylation of α-arrestin Aly1/Art6 at certain sites triggers its ability to promote ubiquitination and downregulation of the Dip5 acidic amino acid transporter (O’Donnell *et al.* 2013). Similarly, glucose promotes dephosphorylation of α-arrestin Rod1/Art4, releasing Art4 to interact with Rsp5 and promote the ubiquitination and endocytosis of hexose transporters at the PM (Becuwe *et al.* 2012; Hovsepian *et al.* 2017). Herrador *et al.* (2015) identified multiple casein kinase phosphorylation sites in the hinge region of the N-terminal arrestin domain of Rim8, and investigated the role of these sites in the RIM pathway. Interestingly, they found that the dephosphorylated Rim8 was constitutively active in RIM pathway signaling, but that the phosphorylation itself appeared to be independent of extracellular pH. Herrador *et al.* (2015) have reported that Rim8 presents as three to four distinct bands that represent different phosphorylated and ubiquitinated species, but the exact distribution of phosphorylated forms in each band has not been elucidated. We have visualized Rim8 in wild-type and *vma2*∆ mutants, but we see little difference in their electrophoretic mobility by SDS-PAGE (data not shown). Further experiments will be necessary to dissect the exact phosphorylation sites on Rim8 under different conditions, their relevance to the RIM and Pma1 internalization pathways, and their susceptibility to CN- and/or Glc7-mediated dephosphorylation.

One difference between Pma1 endocytosis and other α-arrestin-mediated pathways of permease internalization is that the extent of Pma1 endocytosis must be tightly regulated to retain sufficient PM Pma1 for viability. Highlighting the importance of this balance, the synthetic lethality of the *pma1-007* and *vma2*∆ mutations (Figure 7) may arise from further depletion of already reduced Pma1 levels at the PM. Given the fact that too much internalization of Pma1 is lethal, it is attractive to propose that a Pma1 subpopulation is designated, possibly by dephosphorylation, for ubiquitination and endocytosis upon loss of V-ATPase activity. Several sites of phosphorylation have been reported in the cytosolic N- and C-terminal domains of Pma1 that could be targeted by Glc7/Reg1 and/or CN to designate a subpopulation for endocytosis (Goossens *et al.* 2000; Eraso *et al.* 2006; Lecchi *et al.* 2007; Mazon *et al.* 2015). Glc7 reverses Ptk2-generated phosphorylation on S899 of the Pma1 C-terminal tail (Mazon *et al.* 2015), although it is not clear whether Reg1 is required for this step. Pma1 phosphorylation on S899 occurs in the presence of glucose and contributes to enzyme activation, while Glc7 reverses the S899 phosphorylation and activation when glucose is scarce (Mazon *et al.* 2015). If dephosphorylation of S899 were the sole role of Glc7 in signaling Pma1 downregulation, then we hypothesized that we might bypass Glc7 by preventing Pma1 phosphorylation in a *ptk2*∆ mutant. However, we saw no suppression of *glc7-12 vma2*∆ growth defects in a *ptk2*∆ mutant (Figure S1). This indicates that dephosphorylation of Pma1 S899 by Glc7 is not the essential role of Glc7 in generating Pma1 ubiquitination and endocytosis. However, there are other phosphorylated residues in the Pma1 C-terminal tail that could still be important in designating a population for ubiquitination and downregulation. Specifically, Pma1 S911 phosphorylation has been implicated in glucose activation, but neither the kinase responsible, nor the phosphatase responsible for dephosphorylation, has been identified (Lecchi *et al.* 2007; Mazon *et al.* 2015). Interestingly, a study that isolated ubiquitinated peptides from the yeast cell proteome and determined whether they were also phosphorylated (Swaney *et al.* 2013) identified the C-terminal K916 as a potential Pma1 ubiquitination site. The highest level of K916 ubiquitination was observed in nonphosphorylated C-terminal peptides, although some K916 ubiquitination was found in combination with phosphorylated S911 and T912 (Swaney *et al.* 2013). Although these experiments were conducted under conditions where the V-ATPase is active, they suggest interdependent patterns of modification that could be enhanced when organelle acidification is lost.

Consistent with our previous data suggesting that Pma1 internalization is an essential compensatory pathway in *vma* mutants (Smardon and Kane 2014), we found that the introduction of mutations that block Pma1 endocytosis—including *cnb1*∆, *reg1*∆, and *glc7-12*—into the *vma2*∆ mutant caused severe synthetic negative growth phenotypes. The nature of this compensatory effect is not clear. If the compensatory role of Pma1 endocytosis were simply to reduce Pma1 levels at the PM, we anticipated that reducing Pma1 levels via the promoter mutation *pma1-007* would bypass the need for Pma1 endocytosis. However, the *pma1-007* mutation does not suppress the requirement for Rim8-mediated endocytosis in the *vma2*∆ mutant (Figure 7). These results indicate that not only do cells fail to actively sense the level of Pma1 protein at the PM, but internal Pma1 may even help to compensate for loss of V-ATPase activity. Internalized Pma1 ends up in the vacuolar interior where it is degraded, and there is no increase in the vanadate-sensitive ATPase activity (characteristic of Pma1) in vacuoles isolated from *vma* mutant cells (Martinez-Munoz and Kane 2008). In addition, there is no acidification of vacuoles in the *vma* mutants upon glucose addition, suggesting that Pma1 does not contribute to vacuolar acidification (Martinez-Munoz and Kane 2008). It is likely that Pma1, like other membrane proteins destined for vacuolar degradation, enters the interior of the multivesicular body prior to its arrival at the vacuole. However, it is possible that Pma1 could provide some acidification of early endocytic compartments, such as the early endocytic compartment that accumulates in a *rcy1*∆ mutant (Figure 7C), and that this could help account for the compensatory effect. Another alternative is that the mild cytosolic acidification seen in *vma* mutants passively promotes acidification of organelles such as early endosomes. There are currently no probes available that provide ratiometric pH measurement of yeast endosomes; but, using a ratiometric probe of Golgi pH, we observed that as cytosolic pH decreased in the *vma* mutants, Golgi pH also decreased, despite the lack of functional V-ATPases (Tarsio *et al.* 2011). In both of these models, the cell would kill part of the tight cytosolic pH control provided by Pma1 to partially compensate for the loss of organelle acidification.

## Supplementary Material

Supplemental material is available online at www.genetics.org/lookup/suppl/doi:10.1534/genetics.117.300594/-/DC1.
