# Supplementary figures and images for: Compensatory Internalization of Pma1 in V-ATPase Mutants in Saccharomyces cerevisiae Requires Calcium- and Glucose-Sensitive Phosphatases
Source: Genetics. 2017 Dec 18;208(2):655–72. doi: 10.1534/genetics.117.300594 (PMC5788529; doi:10.1534/genetics.117.300594)

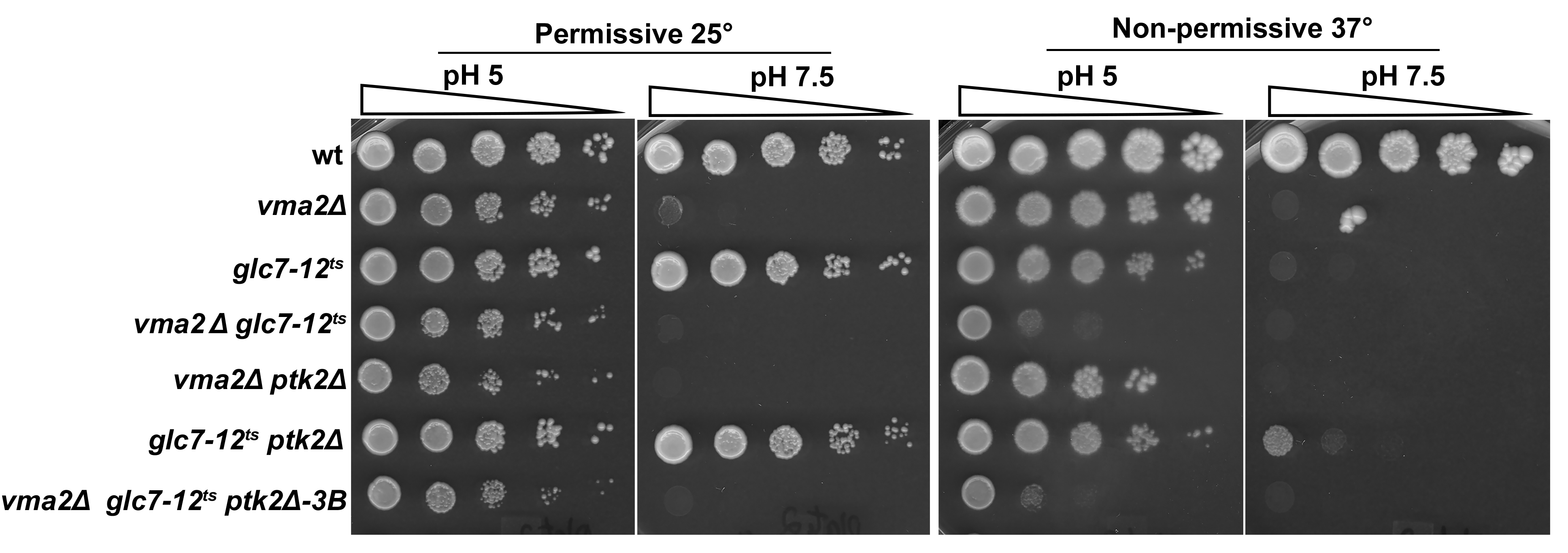

Supplement: Supplementary file 1 [file 655FigureS1.tif]

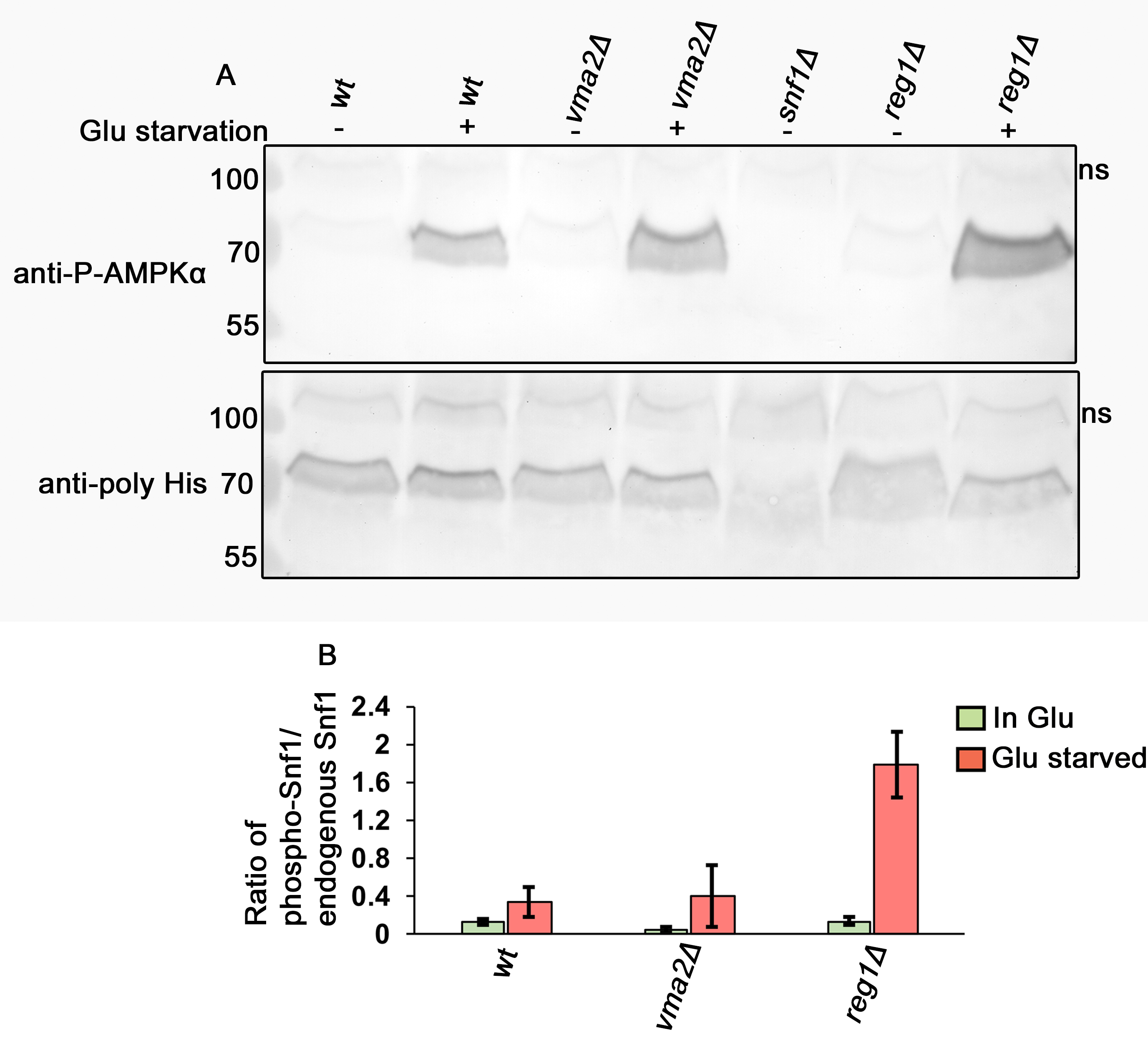

Supplement: Supplementary file 2 [file 655FigureS2.tif]
